# Single cell immune profiling of dengue virus patients reveals intact immune responses to Zika virus with enrichment of innate immune signatures

**DOI:** 10.1371/journal.pntd.0008112

**Published:** 2020-03-09

**Authors:** Yujiao Zhao, Matthew Amodio, Brent Vander Wyk, Bram Gerritsen, Mahesh M. Kumar, David van Dijk, Kevin Moon, Xiaomei Wang, Anna Malawista, Monique M. Richards, Megan E. Cahill, Anita Desai, Jayasree Sivadasan, Manjunatha M. Venkataswamy, Vasanthapuram Ravi, Erol Fikrig, Priti Kumar, Steven H. Kleinstein, Smita Krishnaswamy, Ruth R. Montgomery

**Affiliations:** 1 Department of Internal Medicine, Yale School of Medicine, New Haven, Connecticut, Untied States of America; 2 Department of Genetics, Yale School of Medicine, New Haven, Connecticut, United States of America; 3 Department of Pathology, Yale School of Medicine, New Haven, Connecticut, United States of America; 4 Program in Human Translational Immunology, Yale School of Medicine, New Haven, Connecticut, United States of America; 5 Department of Neurovirology, The National Institute of Mental Health and NeuroSciences (NIMHANS), Bangalore, India; 6 Apollo Hospital, Bangalore, India; 7 Program in Computational Biology and Bioinformatics, Yale School of Medicine, New Haven, Connecticut, United States of America; La Jolla Institute for Allergy and Immunology, UNITED STATES

## Abstract

The genus Flavivirus contains many mosquito-borne human pathogens of global epidemiological importance such as dengue virus, West Nile virus, and Zika virus, which has recently emerged at epidemic levels. Infections with these viruses result in divergent clinical outcomes ranging from asymptomatic to fatal. Myriad factors influence infection severity including exposure, immune status and pathogen/host genetics. Furthermore, pre-existing infection may skew immune pathways or divert immune resources. We profiled immune cells from dengue virus-infected individuals by multiparameter mass cytometry (CyTOF) to define functional status. Elevations in IFNβ were noted in acute patients across the majority of cell types and were statistically elevated in 31 of 36 cell subsets. We quantified response to in vitro (re)infection with dengue or Zika viruses and detected a striking pattern of upregulation of responses to Zika infection by innate cell types which was not noted in response to dengue virus. Significance was discovered by statistical analysis as well as a neural network-based clustering approach which identified unusual cell subsets overlooked by conventional manual gating. Of public health importance, patient cells showed significant enrichment of innate cell responses to Zika virus indicating an intact and robust anti-Zika response despite the concurrent dengue infection.

## Author summary

Mosquitoes carry many globally important human pathogens including a family of related viruses: dengue virus, West Nile virus, Yellow Fever virus, and recently of critical significance, Zika virus. The Zika virus epidemic emerged very rapidly in the susceptible South American population and in many cases immune responses were unable to control the infection. Immune history is a key element of susceptibility or resistance to severe disease. We examined whether pre-existing infection would skew or divert immune resources and might play a role in the severity of Zika infection in the Americas. Using samples from dengue patients and healthy controls from India, we tested functional responses to Zika virus in the context of pre-existing dengue infection. We quantified frequency and functional status of 36 individual cell subsets in depth using advanced profiling techniques and a novel deep learning algorithm. We showed an intact response to new infection with Zika virus which was enriched for early innate immune pathways and robust even during existing dengue infection. Thus, our study suggests that concurrent dengue infection would not be expected to impair immune responses to new infection with Zika virus.

## Introduction

The genus Flavivirus contains many mosquito-borne human pathogens of global epidemiological importance, including dengue virus, West Nile virus (WNV), Yellow Fever virus, and is currently of critical significance with the recent outbreak of Zika virus [1–5]. Dengue has an estimated incidence of 50–100 million infections annually [6–9] and can lead to severe febrile illness with fever, headaches, joint pain, with the most severe manifestations—hemorrhagic fever and shock syndrome—occurring upon a second infection with any distinct serotype. Notably, in endemic regions, seroprevalence levels reach 57% of the population with considerable heterogeneity in clinical symptoms [10]. Similarly, for infections with WNV, which is estimated to have infected 7 million people in the USA [11, 12], the predominate infection outcome is asymptomatic with CDC reporting infection of >46,000 people and more than 2,000 fatalities [12–18]. The closely related Zika virus, first identified in Uganda in 1947 [19], has recently expanded to South America leading to widespread infection including Guillain-Barré syndrome and more than 6,700 cases of microcephaly and neurological abnormalities in newborns [20–25]. As for the other flaviviruses, the majority of infected individuals are asymptomatic or develop mild disease, however Zika virus has been shown to infect fetal brains and neurons and lead to cell death and microcephaly [26–29]. Evidence from pregnant women with acute Zika virus infection suggests that the virus is not always transmitted to the fetus and that only a subset of infants from infected mothers develop detectable neurologic abnormalities.

Disease severity and outcome result from individual differences that define immune status and contribute to responses to infection. Clinical outcomes to viral infection reflect a summary of differences in exposure, genetics (pathogen or host), infection and vaccination history, and acute status. The role of human genetic factors in susceptibility to flavivirus infection includes racial/ethnic differences in susceptibility to dengue [30, 31], single nucleotide polymorphisms in molecules associated with viral entry into susceptible cells or anti-viral immunity [31], as well as epigenetic modifications which accrue over a lifetime [32]. In addition, protection may result from long-term immunity including adaptive T cell responses, neutralizing antibody, and differentiation of natural killer cell responses based on history of viral exposure [33–35].

The rapid emergence of the Zika virus epidemic in previously unexposed populations in South America highlights the possible role of immune history in susceptibility to severe disease. Prior or concurrent infection is a critical element defining immune status and may skew immune pathways or divert immune resources resulting in sub-optimal responses to the subsequent infection. The severity of co-infection is well documented in responses to influenza in aging where infection in older people may be accompanied by severe bacterial pneumonia [36, 37]. In light of the recent epidemic spread of Zika virus infections in dengue virus-endemic areas of Brazil and worldwide in 2015 [25], we conceived of the current studies to assess effects of pre-existing infection with dengue virus on responses to a new viral exposure and whether existing infection might either intensify or curb immune responses to infection with Zika virus. Beyond immune consequences of any co-infections, the similarities of these two viruses are evident—dengue and Zika viruses are closely related genetically and have similar modes of transmission by *Aedes* mosquitoes [5, 38]. Further, recent studies have shown cross-reactivity of dengue-specific immune cells to Zika antigens, higher magnitude of T cell responses to Zika virus following previous dengue infection [39–41] and protection from severe Zika infection by pre-existing humoral immunity to dengue virus [42].

Thus, we reasoned that pre-existing infection might play a role in the severity of Zika infection in the Americas. We assessed this with samples from a cohort of acute dengue patients and controls enrolled in India—which was endemic for dengue but not for Zika at the time of sample collection. These samples provided a unique opportunity to assess effects of pre-existing dengue infection on response to Zika virus. We have investigated functional responses to viral infection in cells from acute dengue patients in comparison to their convalescent samples and to healthy subjects from dengue-endemic areas in India. We employed mass cytometry or CyTOF (Cytometry by Time-Of-Flight) to quantify frequency and functional responses in multiple distinct immune cell populations of the immune system simultaneously and SAUCIE, a novel deep learning algorithm for analysis [43]. These state-of-the-art methodologies for immune profiling [44, 45] provide in-depth characterization of immune mechanisms prevailing in dengue patients and demonstrate that critical elements of initial immune responses to a new viral infection remain intact in the setting of existing dengue infection and would be expected to contribute to resistance to infection with Zika virus.

## Materials and methods

### Ethics statement

Dengue virus patients and healthy volunteers were enrolled with written informed consent under the guidelines of the Institutional Ethics Committee of the NIMHANS (National Institute of Mental Health and NeuroSciences) and Apollo Hospital, Bangalore, India, and Yale University. The Ethics Committees of each institution approved this study.

### Study subjects

Patients with dengue virus infection were classified based on WHO clinical criteria as dengue fever, none as dengue haemorrhagic fever [46]. Dengue infection was confirmed by demonstration of serum dengue NS1 antigen using a Panbio Dengue Early ELISA kit (Inverness Medical Innovations, Australia). Healthy volunteers included household contacts who accompanied the patient to Apollo Hospital and volunteers from the NIMHANS laboratory community. Participants were of both genders (26.7% female) and were all of Indian descent. Subjects from the symptomatic and healthy cohort groups were not statistically different for age, gender, or race in this study (Table 1).

**Table 1:** Demographics of subject cohorts of dengue virus patients and well controls.

Parameter	Dengue acute patients (n = 30)	Well control (n = 15)	Total (n = 45)	*P* value*
**Mean age (yr) (SD)**	28.2 (4.9)	31.3(6.4)	29.2 (5.6)	0.1152
**range**	18–41	24–51	18–51	0.2166
**No. (%) of females**	9 (30.0)	3 (20)	12 (26.7)	

### Sample collection and cell isolation

Heparinized blood was collected from patients, contacts, and healthy volunteers. Purification of peripheral blood mononuclear cells (PBMC) was performed by density-gradient centrifugation using Ficoll-Paque (GE Healthcare) according to the manufacturer’s instructions following isolation and cryopreservation guidelines established by the Human Immunology Phenotyping Consortium [44]. PBMCs for CyTOF were frozen in 90% FBS containing 10% DMSO and stored in liquid N_2_ for shipping following the guidelines of the Department of Biotechnology (DBT), Government of India. Samples for this study were received at Yale in three shipments after receiving authorization for each shipment from the DBT and viability averaged 85% (range 50–98) across the shipments.

### Flow cytometry

The analysis of surface molecules was performed by flow cytometry on fresh cells at NIMHANS. For flow cytometry, monoclonal antibodies from BD Biosciences (CA) were used. Cells were labeled for 30 min at 4°C protected from light with antibodies for surface lineage markers in 8 panels of which 5 were defined by the Human Immunophenotyping Consortium (HIPC) [44] as shown in Supplemental S1A Table. HIPC phenotyping panel marker labeling and detection was performed as described previously [47]. Samples were acquired using a FACS Verse instrument (BD Biosciences, CA) and analyzed using FlowJo software (Tree Star, OR).

### Virus stocks and infection studies

Dengue virus type 2 strain 16681 was propagated in Vero cells (American Type Culture Collection; ATCC, Manassas VA) at 37°C with 5% CO_2_ in DMEM with 10% FBS and 1% Pen/Strep as described previously [48]. For purified virus stock, supernatant was cleared to remove cells and cell debris, and concentrated 100-fold using Centricon centrifugal filter device (100 kda cutoff). Virus stocks were aliquoted, and frozen at -80°C until use. Zika virus strain MEX2-81 was propagated in C6/36 cells (ATCC) at 30°C in DMEM with 10% FBS, 1% tryptose, and 1% Pen/Strep [29, 49]. Zika supernatant was cleared before concentration using a centrifugal filter unit (Millipore #UFC100008). Dengue and Zika virus titers were determined by plaque assay (PFU/ml) in Vero cells as described previously [48] and were 2x10^8^ PFU/ml for dengue and 3x10^9^ PFU/ml for Zika. A single stock of each virus was used throughout the study.

### Virus infection of PBMCs, labeling, and mass cytometry acquisition

For mass cytometry at Yale University, PBMCs (5 x 10^6^ cells/vial) were thawed, incubated in Benzonase (50U/ml) in RPMI/10% human serum, seeded in 96-well culture plate (6 x 10^3^−1.2 x 10^6^ cells/well), and incubated in medium alone or infected with dengue (MOI = 10) or Zika (MOI = 5) virus in vitro for 24h. Experimental infections used a single stock of each virus for infection studies and no difference was detected between uninfected samples incubated in medium alone or in Vero- or C6/36-cell conditioned medium. Monensin (2 μM, eBioscience) and Brefeldin A (3 μg/ml, eBioScience) were added for the final 4 h of incubation for all groups. Groups of samples (8-13/day) were infected in vitro per day on 5 separate days and included a CD45-labeled spike-in reference sample in every sample [50]. Surface markers were labeled prior to fixation and detailed staining protocols have been described [51, 52]. Briefly, cells were transferred to 96-well deep well plates (Sigma), resuspended in 25 μM cisplatin (Enzo Life Sciences) for one minute, and quenched with 100% FBS. Cells were surface labeled for 30 min on ice, fixed (BD FACS Lyse), and frozen at -80°C. Intracellular labeling was conducted on batches of samples (12/day). Fixed PBMCs were permeabilized (BD FACS Perm II) for labeling with intracellular antibodies for 45 min on ice. Cells were suspended overnight in iridium interchelator (125 nM; Fluidigm) in 2% paraformaldehyde in PBS and washed 1X in PBS and 2X in H_2_O immediately before acquisition. A single batch of metal-conjugated antibodies (S1B Table) was used throughout for labeling panels. Metal-conjugated antibodies were purchased from Fluidigm, Longwood CyTOF Resource Core (Cambridge, MA), or carrier-free antibodies were conjugated in house using MaxPar X8 labeling kits according to manufacturer’s instructions (Fluidigm).

A total of 180 samples were assessed by the Helios (Fluidigm) on 15 independent experiment dates using a flow rate of 0.03 ml/min in the presence of EQ Calibration beads (Fluidigm) for normalization. An average of 112,537 ± 71,444 cells (mean ± s.d.) from each sample were acquired and analyzed by CyTOF. The protocol is available at protocols.io (dx.doi.org/10.17504/protocols.io.babdiai6) and the data supporting this study is available at ImmPort (immport.org) under study accession SDY1369.

### Data processing and analysis

All FCS files generated by CyTOF were normalized using Normalizer v0.1 MCR. Manual gating of cell populations and functional markers was performed on the Cytobank platform by exclusion of debris (Iridium^low^, DNA^low^), multi-cell events (Iridium^hi^, DNA^hi^), and dead cells (cisplatin^hi^) as described previously [51] according to standard ontogenies from the Human Immunology Project Consortium [44, 53]. FCS files of live single cells were normalized using SAUCIE, an unsupervised deep learning model that performs several tasks for analysis of CyTOF data including clustering, batch correction, visualization, denoising, and imputation [43]. The batch normalization regularization with MMD was used to perform batch correction to remove technical artifacts but preserve biological variation as described previously [43]. The batch normalized output data was also denoised by imputation on the data manifold by the autoencoder prior to statistical analysis.

## Statistical analysis

Multivariable linear regressions were fit to the outcomes of cell proportions and cytokine expression using predictors of group, stimulation type, and their interaction. Age and gender were included as covariates. A factor capturing within subject covariance across measurements was included, using a compound symmetry structure. To account for the non-normal distribution of cell proportions, we used a generalized linear mixed model assuming a beta distribution and a logit link function. Statistical analysis was performed using the PROC MIXED, PROC GLIMMIX, and PROC LOGISTIC functions of SAS version 9.4 (SAS Institute, Cary, NC).

### SAUCIE automated clustering

SAUCIE information dimension regularization was used to cluster cells into similar cell types for analysis. SAUCIE is based on the autoencoder neural network model that learns to recreate its own input by passing it through a low-dimension informational bottleneck. This bottleneck forces the autoencoder to learn high-level properties of the data that can then be used for further analysis. A two-dimensional bottleneck layer was chosen for SAUCIE, which provided a visualization for analysis of the cell space as shown previously for manually gated T cell subsets [43]. Immune cell lineage markers were assigned as binary variables prior to clustering. By using SAUCIE, each of these tasks was performed on the entire dataset (without dimensionality reduction) or having to employ separate algorithms for visualization, clustering, denoising, or batch correction that may make different (and mutually exclusive) assumptions about the form of the data. Cell proportions derived from the automated clustering were analyzed using a two-part model. First, whether individual observations contributed to a given cluster was modeled with logistic regression. Second, the conditional proportional contributions (contributions > 0) were modeled using the methods described above.

### Enrichment and leading-edge analysis

For each group (acute (n = 30), convalescent (n = 15), well (n = 15)) and stimulation (Zika, dengue), cell/cytokine combinations were ordered by stimulation vs mock p-value (lowest to highest) from the cytokine expression regression analysis. Cell/cytokine combinations with a cell subset from any of the NK, DC, and monocyte subsets (90 combinations out of 360 total) were labeled ‘innate’. The enrichment score (ES) for this innate set was calculated using equation 1 (with p = 0) from Subramanian et al. [54]. Statistical significance of the ES was estimated by generating an empirical null distribution using 100,000 permutations of the cell subset labels for each cytokine. Two-tailed p-values were computed using the percentile method. The leading edge was identified as the cell/cytokine combinations from the start of the ordered list until the ES reached maximal deviation from 0. Analyses were performed using R version 3.6.0 and the tidyverse package [55].

## Results

### Cross platform immune cell profiles of dengue patients

To identify factors associated with susceptibility to flaviviral infection, we enrolled a cohort of study participants with acute dengue infection, and a well cohort comprised of relatives or household contacts of the acute patients and healthy controls from a different endemic site (Table 1). Patients were identified using the WHO criteria [46] and confirmed as dengue-infected by demonstration of NS1 antigen in the serum. Participants were 26.7% female and were all of Indian heritage. To identify characteristic individual differences in immune responses to dengue virus infection, subjects enrolled during acute infection also had a follow-up (convalescent) sample collected after resolution of the acute response (2.5 ± 1.8 months after hospital discharge), at which point functional measurements would be expected to reflect baseline immune status [56, 57].

**Fig 1:**
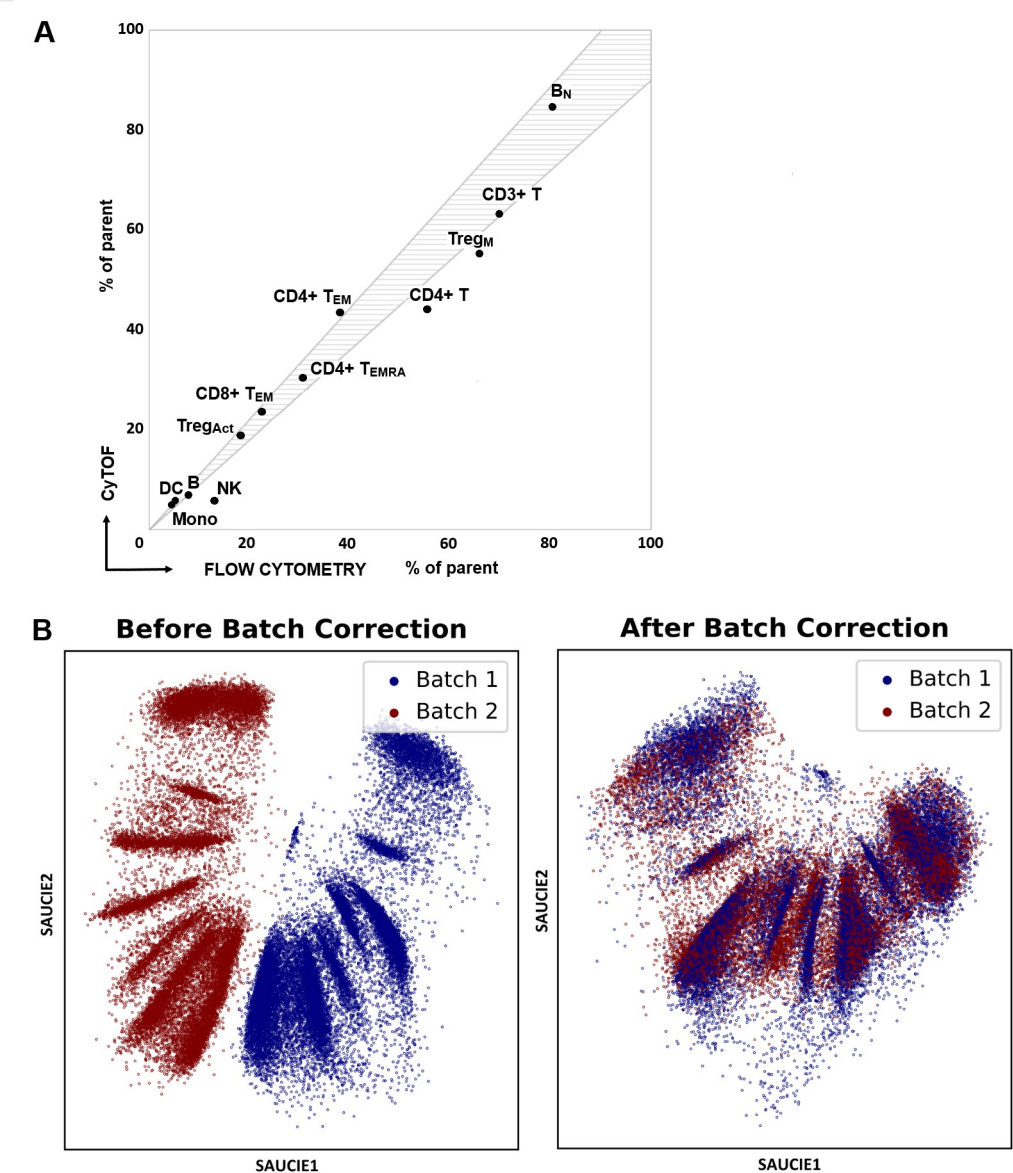
Frequency of immune cell subsets of study cohorts. Study subjects (n = 45) with acute dengue infection, household contacts, and unrelated healthy controls were assessed for frequency of immune cell subsets. PBMCs were profiled fresh by flow cytometry and after cryopreservation by CyTOF. (A) Immune subsets from flow and CyTOF platforms as % of parent cell type (n = 19). Immune subset correspondence between flow cytometry (NIMHANS) and CyTOF (Yale) sites. Shaded area shows 10% variance in the observed distributions. (B) SAUCIE's maximal mean discrepancy (MMD) regularization removes batch effects by penalizing differences in the distributions of the datasets [43]. Shown are two spike-in samples run on different days with a significant batch effect between them. After removal of the batch effects, the two samples are directly comparable.

Samples were collected over a period of 15 months from Apollo hospital (Bangalore). Fresh PBMC were profiled on the day of isolation by flow cytometry using 5 standardized multi-parameter antibody panels of immune markers across 8 fluorescent channels (S1A Table) as recently employed in immunoprofiling a stratified cohort of West Nile virus patients [44, 57]. Cryopreserved samples were shipped to Yale for in depth immune profiling using CyTOF, employing a 40-marker antibody panel to broadly quantify both immune cell lineages and functional markers (S1B Table). Frequencies of immune cell subsets were determined on both platforms after gating according to a standard strategy (S1 Fig). For CyTOF data, markers of functional status within each subset were also quantified simultaneously. Although variability was noted both between individuals and across the cohorts—as would be expected—the frequency of cell subsets within each individual was consistent across both profiling platforms. This congruency was noted despite the differences in cell status—fresh vs cryopreserved—and using different antibody panels and instruments. For example, naïve B cell were present at comparable frequencies (% of parent B cells, flow cytometry 80.24±17.87, CyTOF 84.71±7.99) (Fig 1A). The highly significant degree of correspondence between the subset frequencies determined by two methods at two sites reflects the value of shared protocols in translational investigations with human cells [58]. These consistent results support analysis and interpretation of cellular responses in the study cohort and CyTOF technology was adopted for analysis of samples from India.

### Batch normalization of CyTOF data using neural net autoencoder SAUCIE

Biological variation is a significant challenge to understanding underlying mechanisms in studies with human subjects and model systems. To identify meaningful differences that contribute to diverse clinical responses, experimental variation must be minimized. The current study design includes variations in the days of subject enrollment, shipment to Yale, experimental assay, and instrument run. Numerous measures were adopted to reduce variation in the study as described previously including uniform protocols for sample collection and processing and a single lot of antibody reagents [57, 59, 60]. In addition, a control PBMC sample, prelabeled for CD45, was added to each subject sample as a spike-in reference to minimize the effects of batch variation in CyTOF [50]. Notably, we collected paired samples from acute and convalescent times points (n = 15 dengue-infected patients, 30 samples), and we collected samples at a single timepoint for some acute subjects for whom the convalescent sample was not available (n = 15) and for well subjects (n = 15 relatives/household contacts and unrelated healthy subjects). Each was assessed under 3 culture conditions for a total dataset of 180 samples with 42 quantifiable markers. Processing data of this dimensionality and scale is an inherently difficult prospect, especially considering the degree of noise, batch effects, artifacts, sparsity, and heterogeneity in the data. To assess sources of non-biological variation among samples, we employed a novel unsupervised deep learning model, SAUCIE (Sparse Autoencoder for Unsupervised Clustering, Imputation, and Embedding), that performs several tasks for analysis of CyTOF data including clustering, batch correction, visualization, denoising, and imputation [43]. SAUCIE is based on an autoencoder neural network model that learns to recreate its own input by passing it through a low-dimension informational bottleneck. SAUCIE provides an embedding layer that can be used for visualizing cells in a space that preserves global as well as local information and performs batch correction by regularizing this layer to penalize differences in the distribution of cells from different batches. SAUCIE leverages the ability of an autoencoder to denoise, impute, and visualize, and adds carefully designed regularizations to perform batch correction and clustering, which are essential tasks in single-cell data analysis. Using SAUCIE on the reference (spike-in) cells detected in each experimental sample, we were able to detect equivalence of data collected from samples in each of the shipments and within each in vitro infection day (S2 Fig; differences NS). Notably, CyTOF run day showed statistical difference in the spike-in samples on instrument days, despite instrument performance at expected benchmarks each day of operation. Thus, we used SAUCIE to correct batch effects using Maximal Mean Discrepancy in the CyTOF dataset (Fig 1B).

### Effect of dengue infection on immune cell phenotypic and functional responses

**Fig 2:**
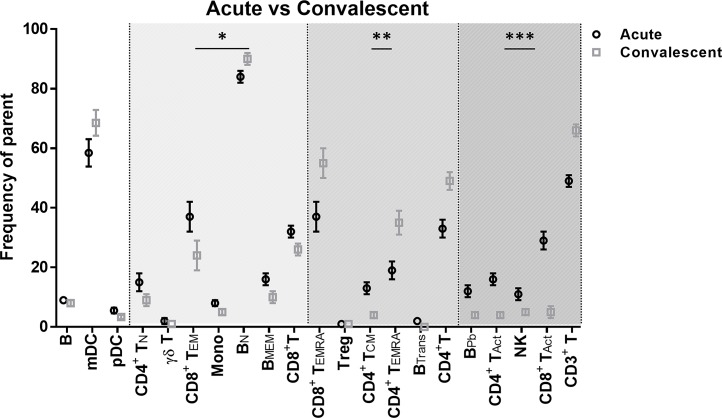
Altered frequency of cell subsets during acute dengue infection. PBMCs from acute patients were labeled with metal-conjugated antibodies and analyzed by mass cytometry. Frequency of cell subsets (percent of parent gate; S1C Fig) between paired samples from patients at acute (○) and convalescent (□) time points (n = 15). Significance assessed by Generalized Linear Mixed Model with * p<0.05, *** p<0.001.

We quantified immune cell subsets of untreated samples by CyTOF following labeling with antibodies for cell lineage specific surface markers (n = 28). Comparing between paired patient samples takes advantage of stable characteristics of individuals to allow a direct comparison at acute and convalescent time points [57, 61]. Differences detected in frequencies of 36 immune cell subsets highlights both similarities and significant differences expected during acute illness and with recovery from infection and may reflect diapedesis into tissue during acute infection (Fig 2; S1C Fig). In particular, in acute patients compared to their convalescent time point, we detected elevated frequencies for monocytes (8.3 ± 1.1 vs 5.3 ± 0.9, P<0.02), activated CD4^+^ and CD8^+^ T cells (CD4^+^16.4 ± 2.4 vs 4.5 ± 1.3, P<0.001; CD8^+^ 29.3 ± 3.3 vs 5.2 ± 1.6, P<0.001), natural killer (NK) cells (11.0 ±1.8 vs 4.6 ± 1.2, p<0.001), transitional B cells (2.3 ± 0.4 vs 0.4 ± 0.2, P<0.001), and plasmablasts (12.3 ± 1.5 vs 4.1 ± 0.9, P<0.001). In contrast, acute subjects showed lower frequencies of subsets including total CD3+ and CD4+ T cells. Such differences in monocytes and expanded populations of CD8+ T cells have also been detected in a previous study of dengue patients [62, 63] and in travelers infected with Zika virus [64, 65]. We note increases in frequency of both CD4+ and CD8+ TEMRA cells at convalescent time points, as has been reported previously in dengue virus donors which may reflect common exposure from an endemic area [66]. Similar differences were noted between frequencies of cell subsets from acute patients compared with well subjects (S3 Fig). Notably, other subsets were not significantly different between acute and well groups, such as CD4^+^ and CD8^+^ TEMRA, γδT cells, memory B cells and monocytes.

**Fig 3:**
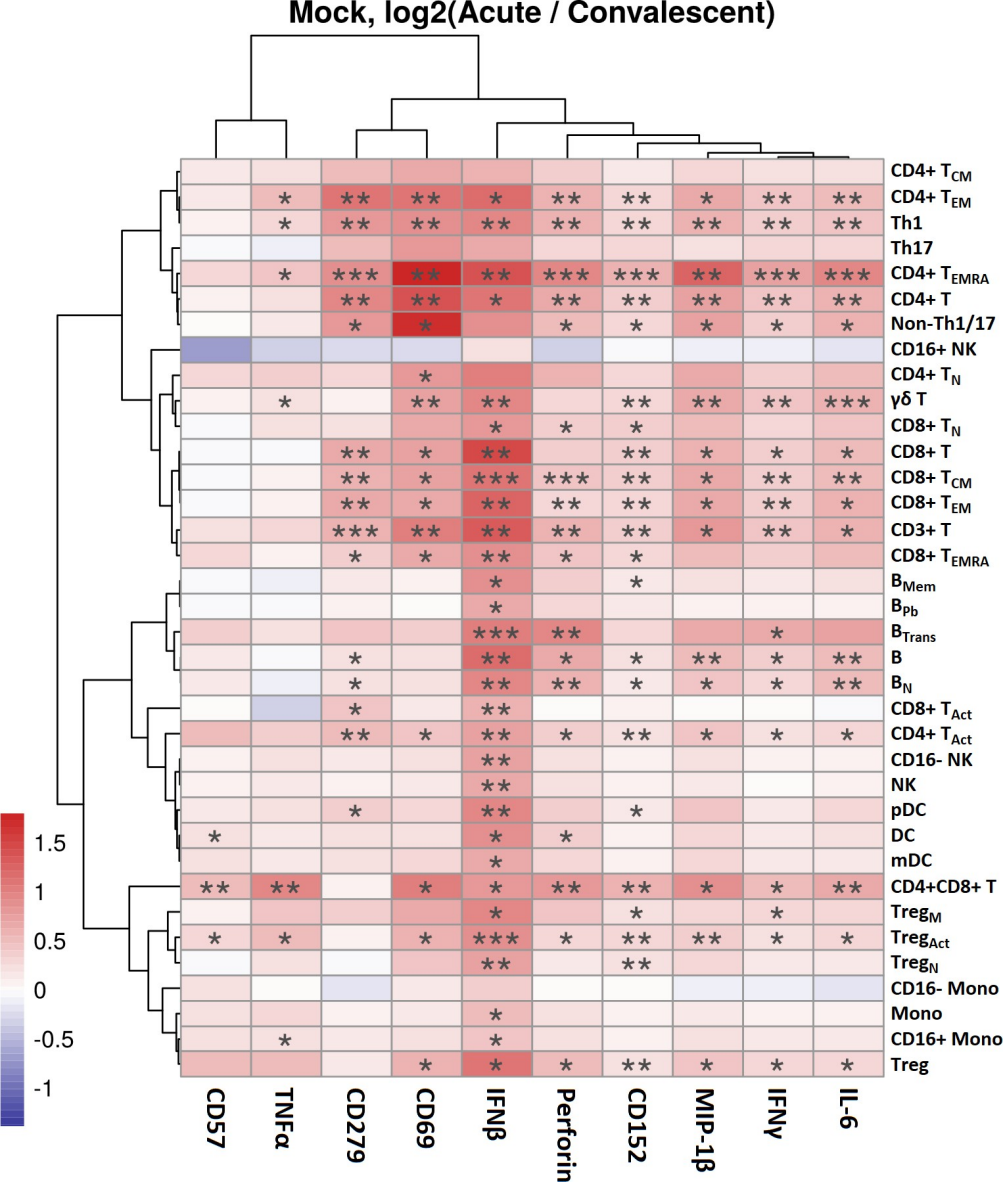
Altered immune functional markers during acute dengue infection. PBMCs from acute patients were labeled with metal-conjugated antibodies and analyzed by mass cytometry. Production of cytokines or changes in activation markers ex vivo (ratio log2 acute/convalescent) was assessed by multivariable linear regression for longitudinal differences between paired samples from patients at acute vs convalescent time points (n = 15). Significance assessed by Generalized Linear Mixed Model with * p<0.05, ** p<0.01 and *** p<0.001.

Cellular activation from viral infection was readily detected ex vivo by elevated levels of key anti-viral markers in baseline, untreated paired samples compared at acute infection and convalescence. In particular, elevations in IFNβ were noted in acute patients across the majority of cell types and were statistically elevated in 31 of 36 cell subsets (S4A Fig) including mDCs, monocytes, NK cells—CD16^+^ and CD16—(Fig 3; P < 0.01), and particularly dramatically increased in cell subsets of plasmacytoid DCs (pDC), B cells (naïve, memory and plasmablasts), and multiple T cell subsets (Fig 3, p<0.05). Elevated levels of IFNβ in plasmablasts have also been found in dengue patients from Thailand [67]. Levels of pro-inflammatory cytokines IFNγ, MIP1β, and IL-6 were higher across T cell subsets of subjects at acute infection compared to convalescence (Fig 3, P<0.05). The active immune response was also reflected in elevated levels of CD279 (PD-1) and CD69 in several T cell subsets (Fig 3, p < 0.05), reflecting activation of those cells. Significant differences were also detected in acute subjects with elevated levels of CD152 (CTLA-4) and perforin in multiple phenotypes of CD4^+^ and CD8^+^ T cells (Fig 3, P<0.05). Further, TNFα and CD57 were significantly elevated in CD4^+^CD8^+^ double positive T cells and activated Tregs (Fig 3, P<0.05). Similarly, the effect of acute illness is evident not only longitudinally in paired samples but in a cross-sectional comparison of markers of functional anti-viral responses from acute subjects compared to well subjects in levels of IFNβ, and other key markers of activation including CD69 and CD279 (S4B Fig). Notably, our in-depth approach revealed high IFNβ levels in one subject from the ‘well’ cohort—likely an incipient infection—however, the differences shown remain significant with or without inclusion of data from that individual. These results (Fig 2, Fig 3) reflect fluctuations in cell subset frequencies and functional markers during acute viral infection consistent with expected changes noted previously [68–70]. In addition, the data provide more in-depth quantitation of cell status during acute viral infection and validate the study samples for in depth functional analysis.

### Effect of existing dengue virus infection on immune responses to new viral challenge

Concurrent infection is a critical element defining immune status and may skew immune pathways or divert immune resources resulting in sub-optimal responses to a subsequent infection. To assess whether active dengue infection or dengue experience (convalescent) impact reinfection of immune cells, we incubated PBMCs ex vivo with dengue virus (reinfection) or Zika virus (new challenge). PBMCs from each subject (acute, convalescent, and well) were infected in vitro with dengue (MOI = 10) or Zika (MOI = 5) virus for 24h and assessed by multiparameter CyTOF as detailed above. As in the assessment of cell status at baseline (Fig 2), we first evaluated cell subset frequency between uninfected (mock) and in vitro stimulated dengue- and Zika-infected samples. We noted equivalent cell frequencies for many cell subsets, including monocyte and DC lineages, as would be expected from innate cell types, as well as for as naïve and memory B cells. Notably, however, in vitro infection with dengue led to a significant increase in frequency of CD4^+^ T cells and subsets of T cells—which was not noted in response to Zika infection—likely reflecting experience with that virus (e.g., Treg, Th2, and CD4+ T_eff_ and CD8+ T_eff_ cells) (S5 Fig). Thus, from the same patients, our results are consistent with an intact T cell memory response for the endemic virus and an apparently primary response for the new viral infection.

**Fig 4:**
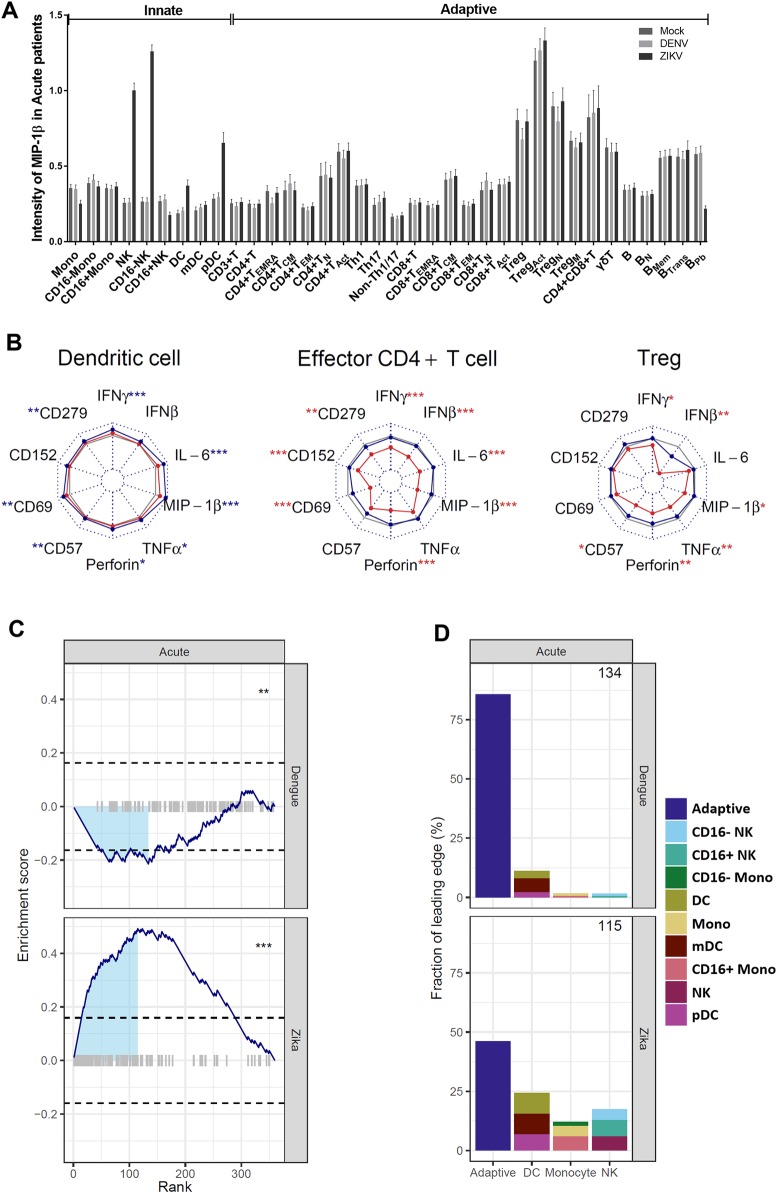
Response to Zika and dengue infection in vitro. PBMCs from acute subjects (n = 30) were incubated with medium alone (mock) or infected in vitro with dengue virus (MOI = 10) or Zika virus (MOI = 5) for 24 h and labeled with metal-conjugated antibodies for mass cytometry. (A) Levels of MIP-1β detected in 36 cell subsets from PBMCs untreated (mock) or stimulated with dengue or Zika virus. (B) For each cell type, proportional changes in 10 activation markers and cytokine levels were quantified comparing mock to the level post-infection with dengue (red lines) or Zika (blue lines) by log2 of the fold-change (infected/mock). The range of plotted fold-changes is indicated at the center and top of each radar plot, with no change (i.e., log2(fold-change) = 0) indicated by the solid grey line. Significant changes are indicated adjacent to each cytokine/activation marker with * p<0.05, ** p<0.01 and *** p<0.001 and color indicates whether the change was significant following incubation with dengue (red *) or Zika (blue *). (C) Each of the 360 cell type/functional marker combinations were rank-ordered by P value (lowest to highest along the x-axis) comparing virus-induced changes with dengue (top panel) or Zika (bottom panel). Enrichment scores for innate cell types were calculated as a running sum (solid blue lines), with comparisons involving innate cell types indicated by grey bars (plotted along the x-axis at an enrichment score of 0). The maximum absolute enrichment score was used to define the “leading edge” (light blue shaded area), and significance of this score was determined by random permutation of cell type labels within each cytokine (horizontal dashed lines indicate the 95% confidence interval). Significance is indicated as: ns (not significant), * p < 0.05, ** p < 0.01, *** p < 0.001. (D) The relative contribution of adaptive and innate cell types to the leading edge of Fig 4C for infection-induced changes with dengue (top panel) or Zika (bottom panel). The number of cell type/functional marker combinations included in the leading edge is indicated in the upper right of each panel.

Incubation in vitro with both dengue and Zika viruses achieved strong activation of the cells from all subjects which was quantified in changes in multiple activation markers and cytokine levels in multiple cell subsets. Using multiparameter CyTOF data allowed us to quantify 10 functional activation markers simultaneously in 36 distinct cell subsets. To examine the possible role of immune history in susceptibility to severe disease, we compared responses in each cell subset by fold change of in vitro treated compared to mock for all acute patient samples (n = 30). We assessed significant differences by a generalized linear mixed model including age and gender as covariates (S2 Table for full data and statistical analysis). A dramatic example is noted in increased levels of the pro-inflammatory cytokine MIP-1β compared to uninfected (mock) cells in numerous cell subsets—in particular from the NK and DC lineages (Fig 4A). Indeed, cell responses reflect abundant changes in vitro noted across the cell types in particular for pro-inflammatory cytokine production (IFNγ, IFNβ, IL-6, MIP1β, TNF0α), and for activation markers CD57, CD69, CD152, CD279. These multivariate data are displayed in a two-dimensional radar plot for representative cell types (Fig 4B). Of the significant differences, some were especially dramatic, including levels across the proinflammatory markers in DCs following incubation with Zika virus where significant increases were detected in IFN, Il-6α, MIP-10β, TNFα, Perforin, CD57, CD69, and CD279 (Fig 4B). Responses of CD4+ T_eff_ cells and Treg cells to dengue virus in vitro showed highly significant differences compared to mock for multiple functional markers as well (Fig 4B). Certain cell responses were equivalently significant in response to in vitro exposure for both viruses, namely CD69 in pDCs, and in mDCs, virus-induced production of cytokines MIP-1β and IFNγ, as well as CD57 and perforin (S2 Table). Unexpectedly, although monocytes are critically important for many flaviviral infections and might be expected to show elevated responses to infection, most monocyte/cytokine combinations did not change significantly after stimulation (p > 0.05; S2 Table).

When we compared the changes in MIP-1β responses, we noted differential responses to the two viruses, with an increase following exposure to Zika virus, and a decrease in response to dengue virus. When we examined this pattern more broadly across other cell types and cytokines/functional markers, we noted a striking pattern of upregulation of responses to Zika infection by innate cell types which was not noted in response to dengue virus. Given our observation of changes in cell frequency between the two viruses where adaptive cell types in vitro specifically tended to change frequency in response to dengue virus (S5 Fig), we sought to determine whether a similar virus-specific pattern was present in the functional activation markers. We quantified the differences vs mock for all significant cell subset/functional marker combinations to assess the most significant changes following virus exposure (S3 Table). For each virus we rank ordered the p-value of the cell type/functional marker combinations differences to calculate an enrichment score of ‘innate’ cell/cytokine combinations using gene set enrichment definitions for cell assignments [54]. For responses to Zika virus, significant differences were detected in functional markers in innate cell lineages in DCs, monocytes, and NK cells for IFNγ, MIP-1β, IL-6, TNFα, with 29/100 significant combinations defined as ‘innate’ and only 4/260 defined as adaptive (S3 Table). Consistent with functional changes following in vitro infection with Zika being significantly enriched for innate cell types, we noted changes in functional markers in adaptive cell types were significantly enriched for dengue. In response to dengue virus, only 5/100 significant differences were defined as innate combinations and 67/260 significant combinations were defined as ‘adaptive’ immune responses. The significant enrichment of innate cell responses to Zika virus indicates that even during acute dengue infection, innate cell types mount an intact and robust anti-Zika response despite the concurrent dengue infection.

To better understand the relative contributions of the different immune cell subsets to the in vitro responses, we compared the composition of significant p-values for innate cell/functional marker combinations and quantified the composition of the leading edge of the enrichment score (Fig 4C). The leading edge following in vitro infection with Zika highlights significant enrichment in innate lineage/functional marker combinations particularly in the DCs, CD16+ monocyte subsets, and NK cell populations, suggesting a broad engagement of the innate response (Fig 4C). The leading edge for dengue is composed mainly (>75%) of adaptive cell types, with subsets of CD4^+^ T cells (activated, effector, effector memory), memory subsets of CD8^+^ T cells, and Tregs expressing elevated levels of CD152, CD69 and producing pro-inflammatory mediators IFNγ, MIP-1β, IL-6, TNFα, and perforin. The relative contribution of adaptive and innate cell types to the leading edge of Fig 4C shows increased adaptive cell types for virus-induced changes with dengue (top panel) and innate cell types for Zika (bottom panel). The difference between responses to Zika virus vs dengue virus is most striking in the acute subjects, however responses to Zika infection were also highly significantly enriched in innate cell types of convalescent and well subjects (S6 Fig). These results of in vitro infections suggest that initial immune responses to Zika infection would be intact and effective in the setting of existing dengue co-infection.

### Novel clustering analysis reveals functional cell populations

**Fig 5:**
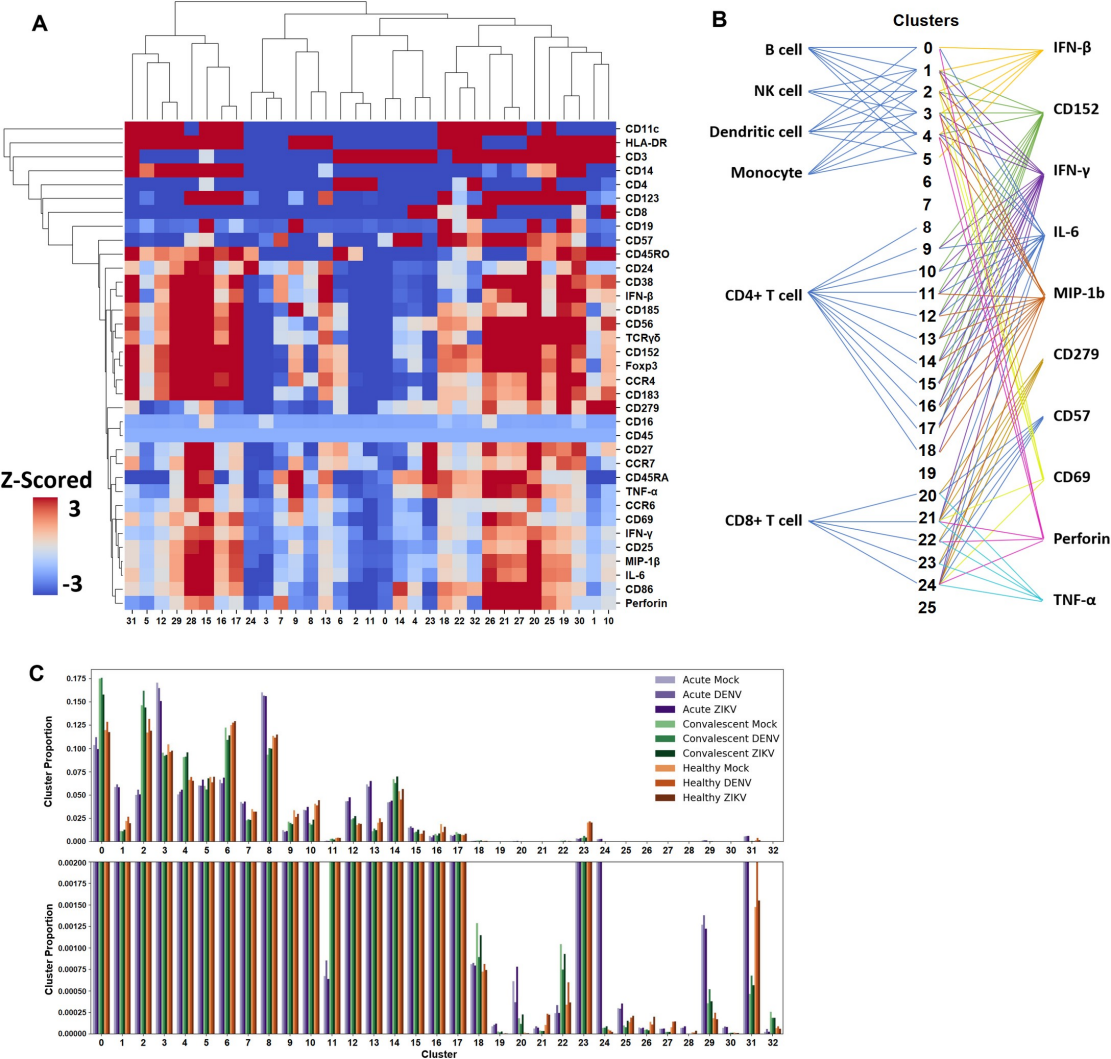
Clustering identifies functional cell populations. Analysis of the clusters obtained from SAUCIE. (A) Heatmap of hierarchical linkage clustering shows relationships between markers. Columns indicating SAUCIE's clusters are shown with rows indicating z-scored mean expression profiles for all markers under analysis. (B). Schematic view of most abundant cell type and markers represented in each cluster for mock and in vitro infected samples of dengue patients and well subjects. (C) A histogram of the proportion of each group's cells that were assigned to each cluster; Inset of smaller clusters with an expanded scale.

The scale and complexity of CyTOF data present challenges for routine analytic approaches. To accelerate analysis, identify additional structure in the CyTOF data, and identify differences among the samples that may be missed by manual gating, we used SAUCIE to cluster the cells using all the CyTOF data from all subjects and all treatments together (Fig 5A). Notably, SAUCIE is scalable—effective with large datasets exceeding 20 million cells—and our analysis includes all cell events collected without need for ‘down-sampling’. SAUCIE directly creates a clusterable representation for each cell by forcing the activations of the network’s internal neurons to be amenable to clustering, thus looking inside the usual “black box” of a deep learning model. Further, SAUCIE reads directly from the data without subjective elements of manual gating which may be prone to error and which have many biases towards pre-conceived canonical cell types. Immune cell lineage markers were assigned as binary variables prior to clustering so that SAUCIE would treat lineage markers differently from functional markers. The assignment of cell types to a cluster offers an unbiased view of cell types and functional markers which may be relevant to understanding of immune status. In depth examination of the markers in SAUCIE clusters suggest that clusters contain elevated proportions of certain cell types and can be characterized by their dominant cellular markers (Fig 5A and 5B). Specifically, SAUCIE clusters 9, 15, 18, 19, and 20 prominently feature B cell marker CD19+ cell types subsets, and SAUCIE clusters 12, 29, 15, 16, 17, and 31 contain the bulk of the monocytes. CD3^+^ T cell subsets are represented in 19 distinct clusters, which reflects the multiple markers in the antibody panel to define distinct T cell subsets. Cluster 32 is populated with both CD4+ and CD8+ T cells. Interestingly, for the CD4+ subpopulations, cluster 25 shows a profile of pro-inflammatory activation expressing IFNγ, MIP-1β, IL-6, CD25, and is low for CD45RA, a marker of memory responses. This is in contrast to CD4+ T cells of cluster 32, which are higher in the naïve T cell marker CD45RA and lower for the effector cytokines, reflecting a more naïve phenotype. For CD8+ T cells, cluster 10 shows very high expression of CD279 (PD-1) and CD45RO, suggesting a memory cell type, distinct from CD8+ T cell cluster 26 with very high expression of effector cytokines and perforin.

We quantified the proportion of each cohort and stimulation condition in each cluster and found dramatic differences between frequencies in different subject groups in our study (Fig 5C). These findings from SAUCIE clustering agree with manual gating and statistical evaluation of cell subsets. We examined clusters which were statistically significantly elevated in samples from acute patients compared to convalescent or well, which we expect to include markers relevant to the initial response to the viral infection. By analyzing the channel intensity of markers, we found that samples from acute subjects showed the highest representation in clusters 1, 3, 7, 8, 12, 13, 29, 31 which express CD14, CD4, CD56, TCRγδ, and proinflammatory cytokines with IFNβ and perforin. Cluster 29 also includes markers CD38, CD24, and CD185 (CXCR5) suggesting transitional/immature B cells, as would be expected during acute infection as the antibody response is developing [71] and expression of functional molecules, i.e., IFNβ, chemokine receptors CXCR5 and CCR6, relevant for function of the 4 critical subsets predominant during acute infection. Samples from acute subjects have the lowest representation in clusters 0, 2, 4, 6, and 11 in which the dominant markers are CD3, CD4, CD45RO and CCR7, suggesting a phenotype of activated memory T cells which increase in the convalescent group (Fig 5C). Cluster 31, highly expressed in acute samples, includes elevated expression levels of many of the innate markers in their nearest neighbor, cluster 5, but also show CCR4, CCR6, CD183, TCRγδ, CD185. Cluster 31, which lacks CD4 and CD8, is the farthest apart on the heat map from cluster 10, which expresses CD8 very highly. Thus cluster 31 may indicate in part anti-flaviviral responses including TCRγδ cell population, a major early source of IFNγ [72]. Cluster 19, a smaller cluster which is not significantly different between subject groups, has expression of multiple lineage markers and effector molecules IFNβ and perforin indicative of active NK cells (CD56, CD38, perforin) and memory T cell response (CD45RO, CD8, CD279) [73]. A number of clusters (19–32), which are lower in abundance, show high representation of cell markers CD3, CD45RO, IL-6, Foxp3, CD152, indicating memory T cells with regulatory pathways engaged to reduce cytokine production, as would be expected in the post-viral recovery period.

A notable feature of SAUCIE derives from the unbiased data-driven reading directly from the data. In addition to findings consistent with manual gating, SAUCIE also offers additional, more granular information on cell populations that were not isolated via manual gating. For example, SAUCIE identified novel clusters not identified using manual hierarchical gates which may reflect relevant transitions in the acute immune response. These “silent” cells are recognized by SAUCIE as a distinct cluster of cells and represent 12% of total cells yet are outside of any manual gate. Such ‘silent cells’ are overlooked by the traditional manual gating strategies noted in S1 Fig. Specifically, cluster 0, which includes cells positive almost exclusively for CD3, which statistical comparison finds is lower in acute subjects than convalescent samples (P< 0.001), suggests few of these CD3-only T cells would be present during acute infection. Further, SAUCIE identified cells in a unique cluster 3 which includes cell types lacking strong expression of any lineage markers. Cells in cluster 3 are highly elevated in acute time points compared to convalescent or well subjects (P< 0.001), suggesting a transient population of possibly immature or less committed cells which are evident during an acute response. Such cell subsets were revealed using SAUCIE, confirmed by manually gating, and introduce novel elements for interpretation and evaluation of cellular responses. Taken together, the clusters identified by SAUCIE provide an unbiased mechanism to view the complex data generated from in-depth immune profiling studies across multiple human subjects in distinct subject groups.

## Discussion

Significant advances in technology—including single cell platforms—have driven the field of Systems Immunology, where data-driven approaches provide insights for translational studies [74, 75]. Human immunoprofiling has recently provided critical advances in our understanding of responses to influenza and malaria vaccines [76–78]. Such systems approaches are designed to build on stable characteristics of individual immune cell profiles [57, 61, 79] to define molecular interactions and advance mechanistic understanding which can be employed to promote effective immunity.

The current study is based on an effective international collaboration between investigators in the USA and India, which is endemic for dengue infection, and builds on similar systems level work by our group to identify a susceptibility signature from a stratified cohort of West Nile virus patients [57]. To address critical elements of immune responses that may contribute to susceptibility to Zika virus infection, we have investigated a role for underlying infection with dengue, which is endemic in South America and carried by the same *Aedes* mosquito [5, 38]. Here we have quantified functional immune profiles of multiple arms of the immune system and assessed responses between patients with dengue infection (acute or convalescent) compared to an uninfected control cohort from the same endemic area. We hypothesized that existing infection with dengue might intensify or curb immune responses to infection in vitro with Zika, and thus be relevant for the epidemic. While this 24 hour ex vivo design cannot address more complex responses in vivo, results from initial responses suggest efficient immune responses across multiple cell types. Notably, our data show that innate immune responses to Zika infection are engaged in the setting of existing dengue infection and thus pre-existing infection with dengue virus would not be expected to impair responses to new infection with Zika virus. However, our studies do not address whether the activation of innate cells noted in vitro would contribute to clearance of virus. Other recent studies with Zika virus have noted downregulation of antiviral gene pathways, particularly interferon responsive genes [80, 81] and inhibition of NK cell killing pathways [82], both key elements of host ability to respond to infection.

Investigations in both human and murine hosts show that adaptive immune responses to primary dengue infection influence the course of subsequent severe disease, as the primary weak broadly neutralizing antibodies developed may promote antibody-dependent enhancement (ADE) of subsequent dengue infection in FcγR-bearing cells [83, 84]. In addition, some T cell response patterns during primary dengue infection have been hypothesized to contribute to reduced efficiency of T cell responses in secondary infection, a phenomenon termed ‘original antigenic sin’ [85]. Kinetic studies have shown that the innate responses that accompany early dengue expansion (< 3 days), like production of the proinflammatory cytokine, TNF-α, from infected myeloid cells, do not mediate the later severe effects of secondary dengue infection (i.e. plasma leakage) [6, 86] but may polarize responses relevant to resistance to a new infection. Studies in vaccine development clarify the potential role of pre-existing dengue immune sera, which leads to enhancement of severe infection with dengue virus [84] and through cross-reactivity with Zika [87] may enhance uptake of ZIKA in monocytes [88, 89]. However, while pre-existing immunity to flaviviruses did not alter outcome to infection with Zika in rhesus macaques [90], it has in contrast been protective against lethal fetal demise in mice [91, 92], enhanced responses to specific targets of ZIKA E, prM, and C [39], and resulted in broader cross-reactive antibody repertoire [93] and protection against infection in humans [42].

Using a shared immune profiling approach in two countries, we have documented previously-defined elements of anti-dengue immunity such as elevated levels of activation markers (e.g., CD57 on T cells) and increased production of inflammatory mediators (e.g., IFNβ, perforin, IFNγ) [85]. And importantly, in our studies of subsequent infection in vitro, we show robust immune responses to Zika virus from patient cohorts. The increased proportion of innate inflammatory mediators from acute patients to Zika viruses in comparison to infection with dengue virus may reflect an already maximal response to the concurrent infection which may be expected as cells from acute patients may approach maximal activation during that phase of their illness. While our in vitro studies cannot incorporate all elements of natural infection, and our sample size may be insufficient to detect smaller effects, our study suggests that primary dengue infection would not impair or limit immune responses to infection with Zika. Furthermore, previous studies have reported that cross-reactive peptide sequences led dengue-exposed subjects to develop stronger anti-Zika CD4 and CD8 T cells responses [39–41], suggesting that exposure to dengue infection may in fact augment cellular immune responses to infection with Zika.

Our results are significant for the in-depth profiling of a large dataset and the use of deep learning to reduce effects of experimental variation and enhance analysis of meaningful variation. Our neural network program SAUCIE provides valuable insights into the phenotypic and functional states which distinguish the patient populations. SAUCIE identified cellular responses that were consistent with cells identified through manual gating and traditional statistical approaches and accelerated this identification. In addition, SAUCIE identified a distinct cluster of cells outside any gate and overlooked by manual gating. Further definition of molecular signatures characteristic of flaviviral immunity and susceptibility phenotypes is essential to guide development of improved diagnostics, therapeutic interventions and vaccines for Zika. Investigations to understand the ferocity of the Zika epidemic may include ongoing research areas of epidemiological factors such as co-circulating pathogens [94], cellular signaling pathways antagonizing the Type I or Type II IFN responses [80, 95, 96], or other immune pathways such as RIG-I [97], viperin [98], or pathogenic cellular interactions including CD8 cells in pathology in neurons [99] or monocytes driving NK cells anti-Zika responses [100].

## Supporting information

S1 Fig Representative gating strategy for flow cytometry (A) and CyTOF (B) and subset population definitions defined by hierarchical gating (C).
(TIF)

S2 Fig SAUCIE analysis of experimental batch effects. (TIF)

S3 Fig Altered frequency of cell subsets between acute patients and well subjects. (TIF)

S4 Fig Altered immune functional markers between dengue subjects. (TIF)

S5 Fig Heatmap of equivalence of cell frequency following in vitro infection. (TIF)

S6 Fig Response to Zika and dengue infection in convalescent patients and well subjects. (TIF)

S1 Table Antibody panels used for flow cytometry (A) and CyTOF (B).
(DOCX)

S2 Table Production of cytokines or changes in activation markers in acute subjects. Average median channel values for each functional marker in each cell subset for dengue patients at the acute timepoint (n = 30). Significant differences vs mock for cell-marker combinations (p< 0.05) are highlighted for each virus: dengue orange, Zika blue.
(PDF)

S3 Table Ranked sum of p values for enrichment of cell/activation markers. Columns show p value for differences vs mock for cell subset-activation marker combinations in response to infection with dengue or Zika virus in vitro. P values for dengue patients at acute and convalescent time points and well subjects are shown with differences p<0.05 highlighted in orange.
(PDF)
