# Supplementary figures and images for: Single cell immune profiling of dengue virus patients reveals intact immune responses to Zika virus with enrichment of innate immune signatures
Source: PLoS Negl Trop Dis. 2020 Mar 9;14(3):e0008112. doi: 10.1371/journal.pntd.0008112 (PMC7082063; doi:10.1371/journal.pntd.0008112)

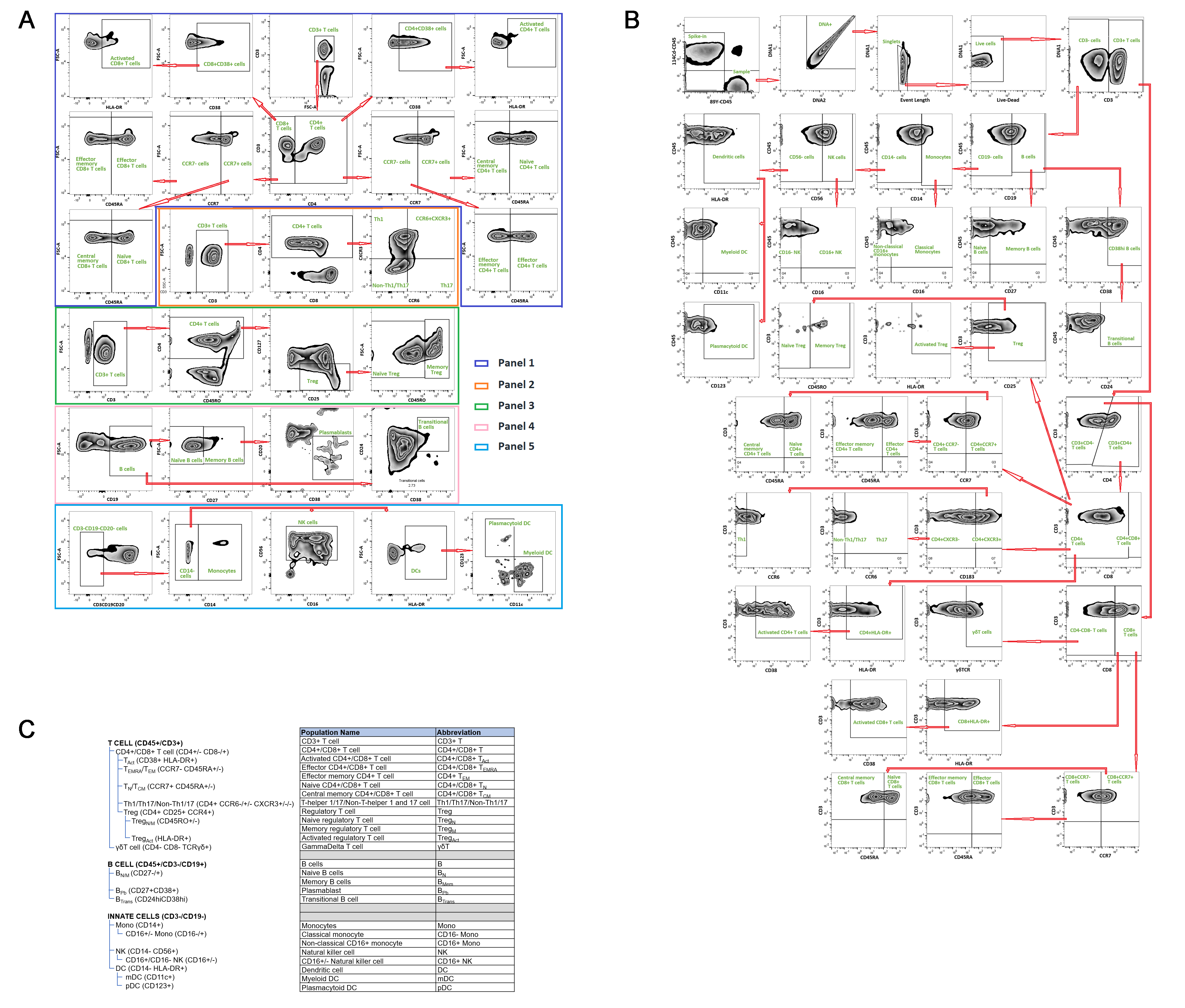

Supplement: S1 Fig — Representative gating strategy for flow cytometry (A) and CyTOF (B) and subset population definitions defined by hierarchical gating (C). (TIF) [file pntd.0008112.s001.tif]

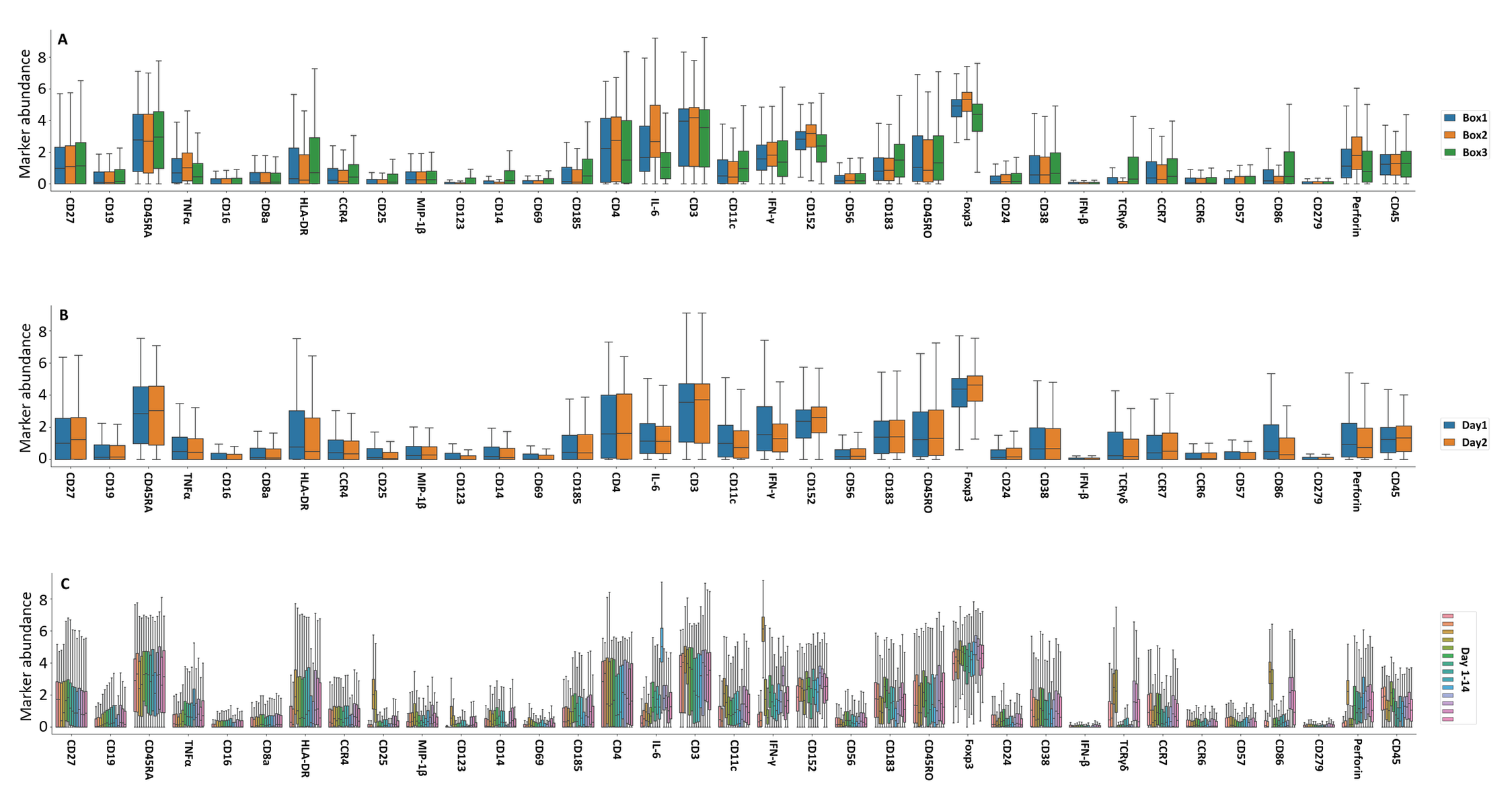

Supplement: S2 Fig — (TIF) [file pntd.0008112.s002.tif]

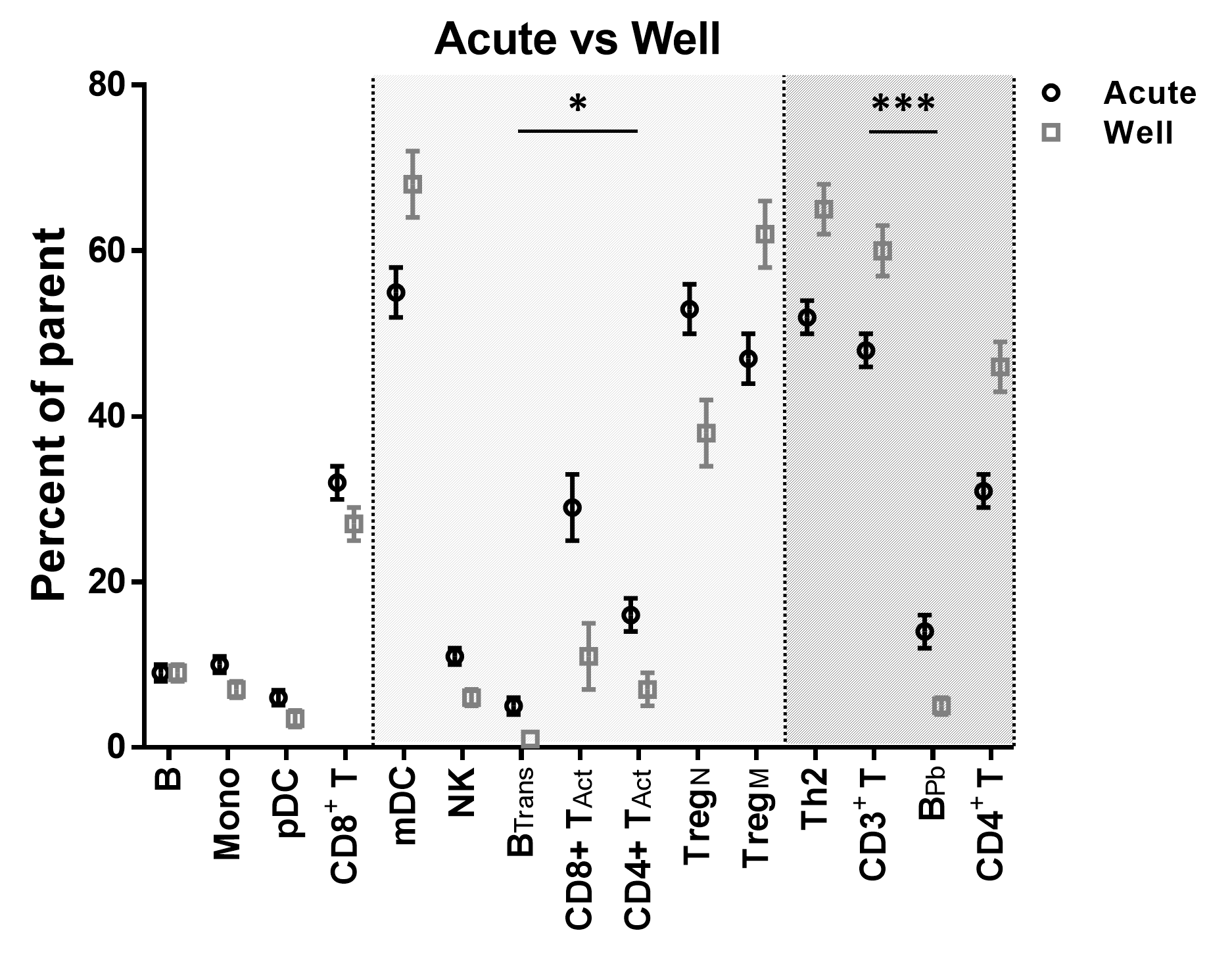

Supplement: S3 Fig — (TIF) [file pntd.0008112.s003.tif]

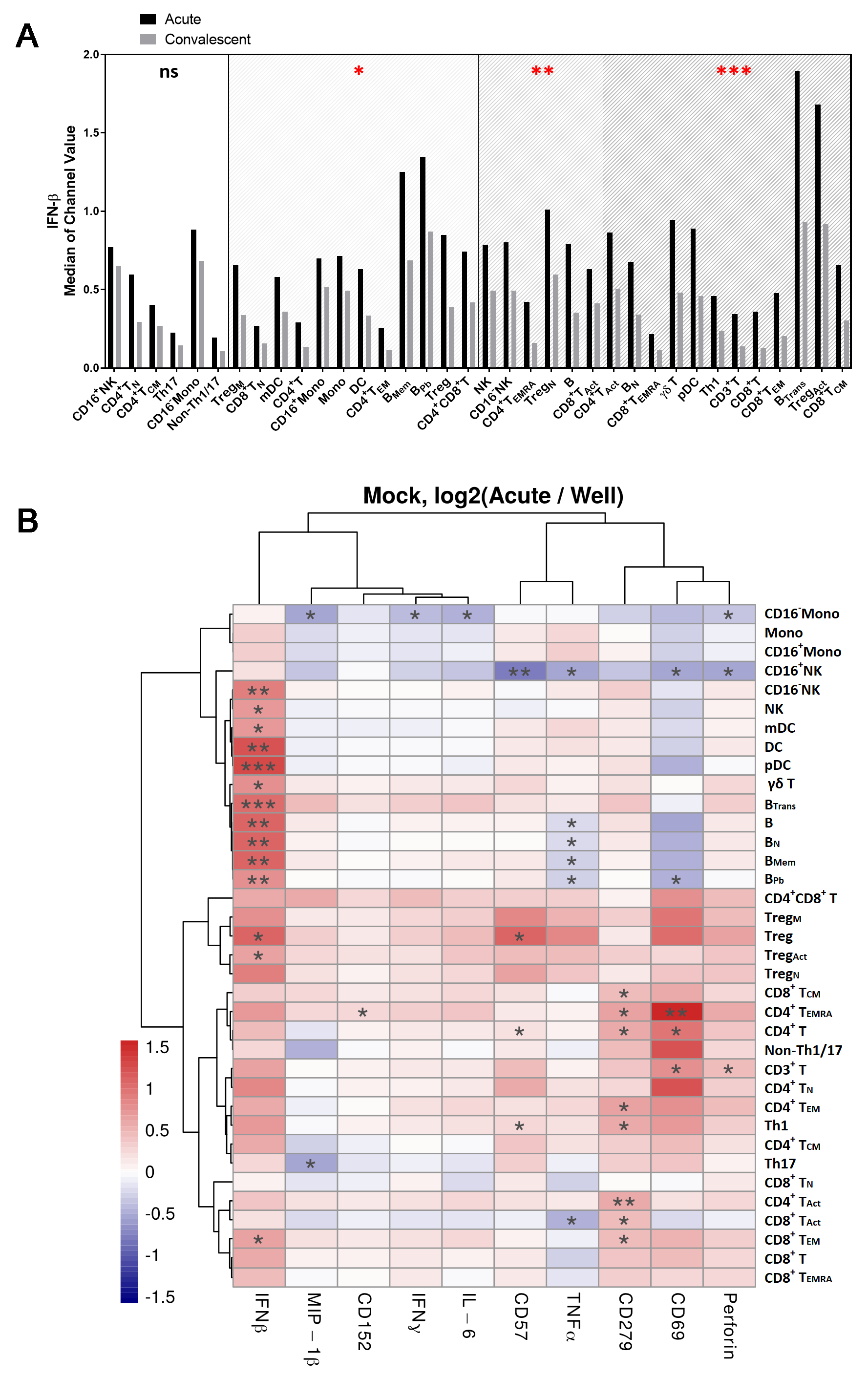

Supplement: S4 Fig — (TIF) [file pntd.0008112.s004.tif]

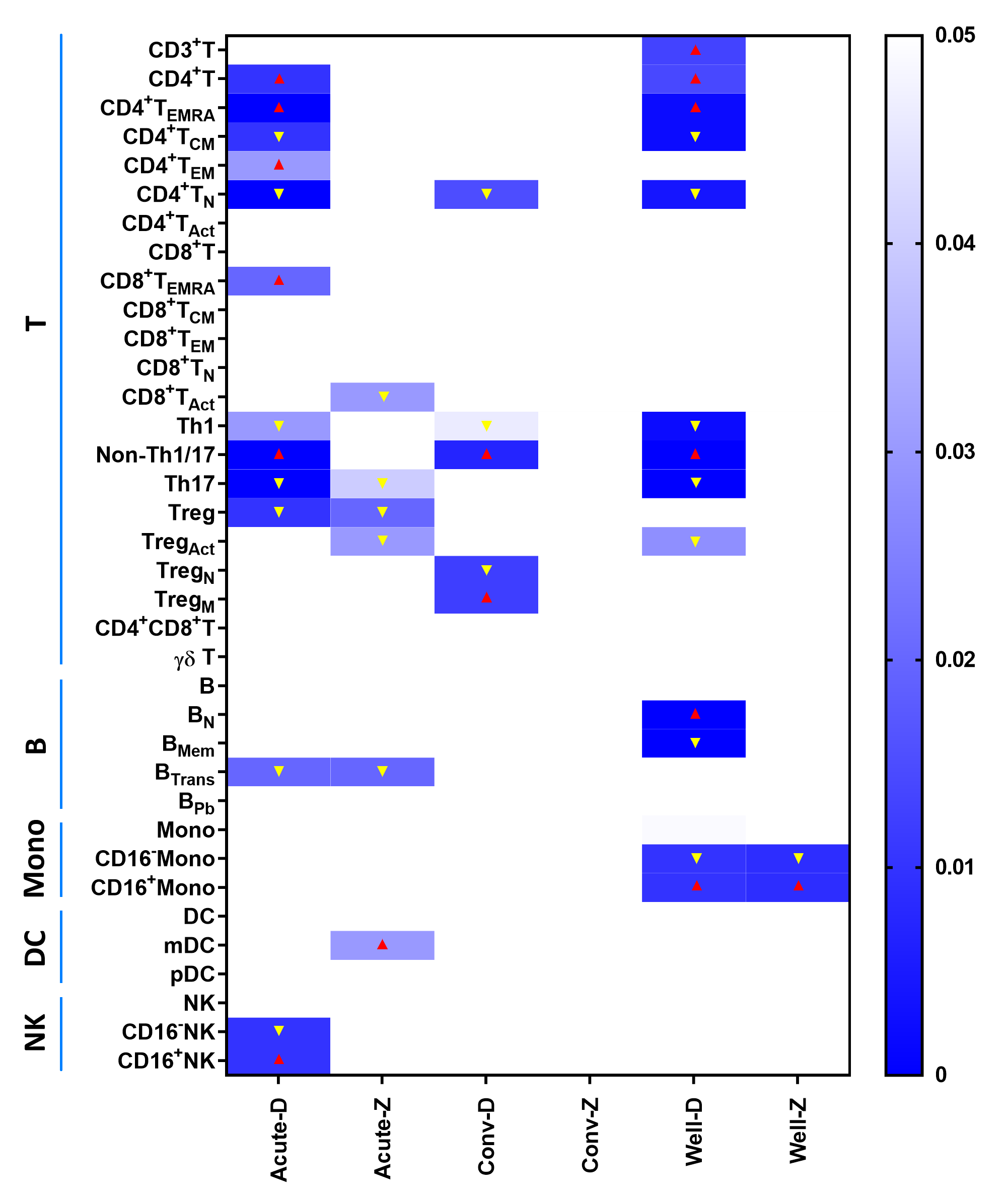

Supplement: S5 Fig — (TIF) [file pntd.0008112.s005.tif]

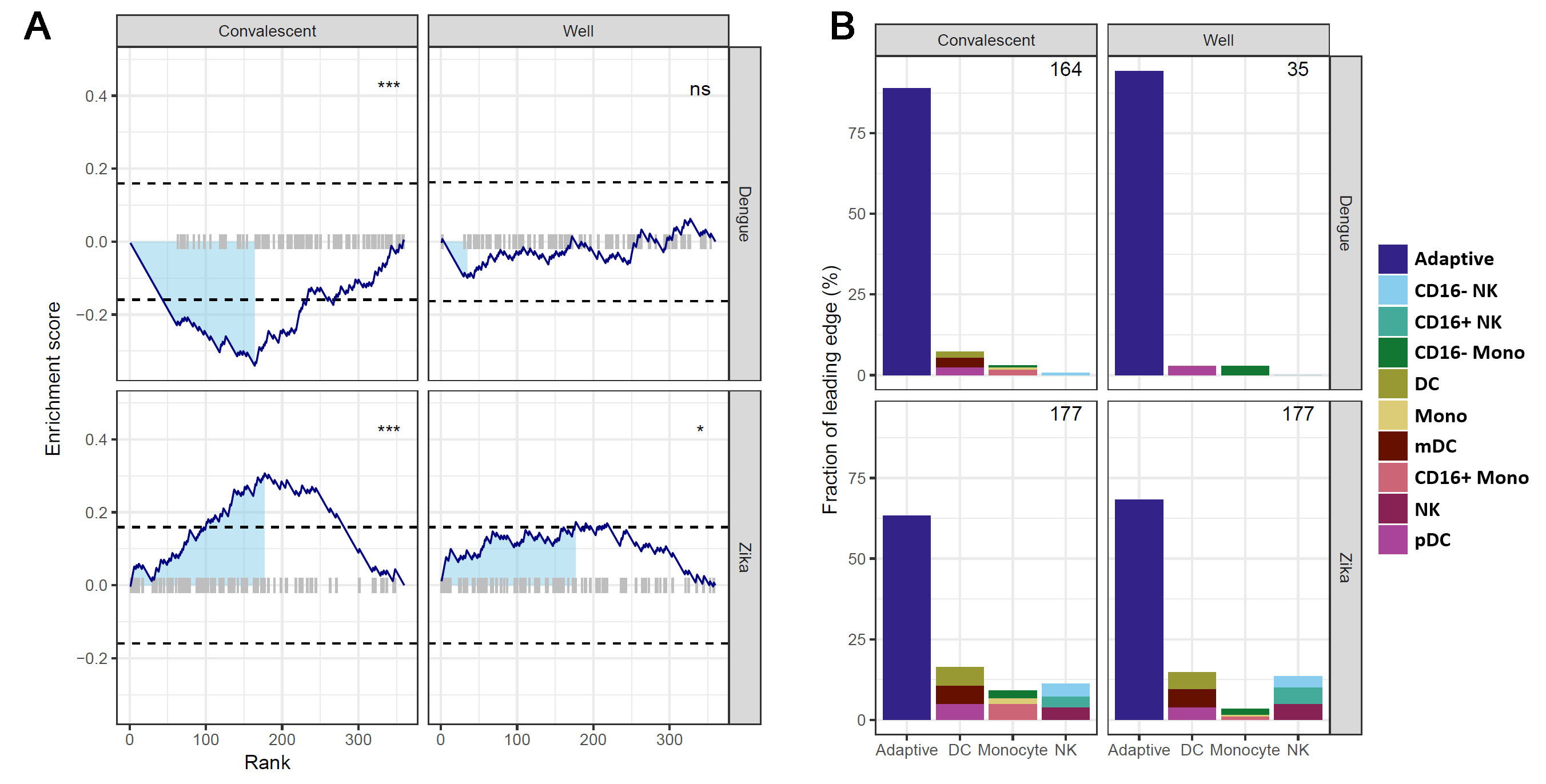

Supplement: S6 Fig — (TIF) [file pntd.0008112.s006.tif]
