# Supplementary material for: Single cell immune profiling of dengue virus patients reveals intact immune responses to Zika virus with enrichment of innate immune signatures
Source: PLoS Negl Trop Dis. 2020 Mar 9;14(3):e0008112. doi: 10.1371/journal.pntd.0008112 (PMC7082063; doi:10.1371/journal.pntd.0008112)
Supplement: S1 Table — Antibody panels used for flow cytometry (A) and CyTOF (B). (DOCX) [file pntd.0008112.s007.docx]

**Table S1A. Antibodies used for flow cytometry (NIMHANS).**

| Fluorophore/Isotope | Ab | Vendor | Catalog # | Clone |
| --- | --- | --- | --- | --- |
| PE | CCR7 | BD | 560765 | 150503 |
| PE | CD25 | BD | 341009 | 2A3 |
| PE | CD24 | BD | 555428 | ML5 |
| PE | CD56 | BD | 555516 | B159 |
| PE | CXCR3 | BD | 557185 | 1C6 |
| PERCP-CY5.5 | CD4 | BD | 341654 | SK3 |
| PERCP-CY5.5 | CD19 | BD | 340951 | SJ25C1 |
| PERCP-CY5.5 | CD123 | BD | 558714 | 7G3 |
| PE-CY7 | CD45RA | BD | 337167 | L48 |
| PE-CY7 | CCR4 | BD | 557864 | 1G1 |
| PE-CY7 | CD27 | BD | 560609 | M-T271 |
| PE-CY7 | CD11c | BD | 561356 | B-LY6 |
| PE-CY7 | CCR6 | BD | 560620 | 11A9 |
| APC | CD38 | BD | 340439 | HB7 |
| APC | CD127 | BD | 560905 | HIL-7R-M21 |
| APC | CD16 | BD | 561304 | B73.1 |
| APC-H7 | CD8 | BD | 560179 | SK1 |
| APC-H7 | CD45RO | BD | 561137 | UCHL1 |
| APC-H7 | CD20 | BD | 560734 | 2H7 |
| APC-H7 | CD3 | BD | 560176 | SK7 |
| APC-H7 | CD19 | BD | 643078 | SJ25C1 |
| V450 | CD3 | BD | 560365 | UCHT1 |
| V450 | CD14 | BD | 560349 | MPHIP9 |
| V500 | HLA-DR | BD | 561224 | G46-6 |
| V500 | IgD | BD | 561490 | IA6-2 |

**Table S1B. Antibodies used for mass cytometry (Yale).**

| Fluorophore/Isotope | Ab | Vendor | Catalog # | Clone |
| --- | --- | --- | --- | --- |
| 89Y | CD45 | Fluidigm | 3089003B | HI30 |
| 141Pr | CD27 | Longwood |  | O323 |
| 142Nd | CD19 | Longwood |  | HIB19 |
| 143Nd | CD45RA | Fluidigm | 3143006B | i100 |
| 144Nd | TNFα | Longwood |  | MAb11 |
| 145Nd | CD16 | Longwood |  | 3G8 |
| 146Nd | CD8a | Fluidigm | 3146001B | RPA T8 |
| 147Sm | HLA-DR | Longwood |  | L243 |
| 148Nd | CCR4 | Biolegend | 359402 | L291H4 |
| 149Sm | CD25 | Longwood |  | M-A251 |
| 150Nd | MIP-1β | Fluidigm | 3150004B | D21-135 |
| 151Eu | CD123 | Biolegend | 502302 | BVD2-21C11 |
| 152Sm | CD14 | Fluidigm | 3152001B | MAb11 |
| 153Eu | CD69 | Beckman Coulter | IM3337 | FES172 |
| 154Sm | CD185(CXCR5) | Longwood | V05525 | J252D4 |
| 155Gd | CD4 | Longwood | V04286 | RPA T4 |
| 156Gd | IL-6 | Fluidigm | 3156011B | MQ2-13A5 |
| 158Gd | CD3 | Longwood |  | UCHT1 |
| 159Tb | CD11c | Fluidigm | 3159001B | Bu15 |
| 160Gd | IFNγ | Longwood |  | 4S.B3 |
| 161Dy | CD152(CTLA-4) | Fluidigm | 3161004B | 14D3 |
| 162Dy | CD56 | Longwood |  | NCAM16.2 |
| 163Dy | CD183(CXCR3) | Fluidigm | 3163004B | G025h7 |
| 164Dy | CD45RO | Fluidigm | 3164001B | W6D3 |
| 165Ho | Foxp3 | Longwood | V03318 | FoxP3 |
| 166Er | CD24 | Fluidigm | 3166007B | ML5 |
| 167Er | CD38 | Fluidigm | 3167002B | O323 |
| 168Er | IFNβ | Longwood |  | IFNb/A1 |
| 169Tm | TCRγδ | Longwood |  | B1 |
| 170Er | CCR7 | Longwood |  | G043H7 |
| 171Yb | CCR6 | BioLegend | 353427 | G034E3 |
| 172Yb | IgM | Longwood |  | MHM-88 |
| 173Yb | CD57 | BioLegend | 322325 | HCD57 |
| 174Yb | CD86 | Longwood |  | IT2.2 |
| 175Lu | CD279(PD-1) | Fluidigm | 3175008B | EH12.2H7 |
| 176Yb | Perforin | Longwood |  | dG9 |
| Q-dot | CD45 | Longwood |  | HI30 |
