# Supplementary material for: Single cell immune profiling of dengue virus patients reveals intact immune responses to Zika virus with enrichment of innate immune signatures
Source: PLoS Negl Trop Dis. 2020 Mar 9;14(3):e0008112. doi: 10.1371/journal.pntd.0008112 (PMC7082063; doi:10.1371/journal.pntd.0008112)
Supplement: S2 Table — Average median channel values for each functional marker in each cell subset for dengue patients at the acute timepoint (n = 30). Significant differences vs mock for cell-marker combinations (p< 0.05) are highlighted for each virus: dengue orange, Zika blue. (PDF) [file pntd.0008112.s008.pdf]

**Table S2. Production of cytokines or changes in activation markers**

| | | CD152 | | | CD279 | | | CD57 | | | CD69 | | | IFNb | |
|---|---|---|---|---|---|---|---|---|---|---|---|---|---|---|---|
| | cell | Mock | DENV | ZIKV | Mock | DENV | ZIKV | Mock | DENV | ZIKV | Mock | DENV | ZIKV | Mock | DENV |
| Innate | Mono | 1.540979 | 1.518649 | 1.579467 | 0.204847 | 0.202093 | 0.210597 | 1.112414 | 1.096952 | 1.110568 | 0.300018 | 0.298567 | 0.311071 | 0.673975 | 0.642393 |
| | CD16$^-$Mono | 1.649076 | 1.631341 | 1.653793 | 0.220457 | 0.228805 | 0.226287 | 1.226494 | 1.248829 | 1.225731 | 0.355954 | 0.353071 | 0.350461 | 0.71921 | 0.700401 |
| | CD16$^+$Mono | 1.536197 | 1.517301 | 1.579598 | 0.204781 | 0.199383 | 0.210521 | 1.101469 | 1.079429 | 1.097321 | 0.29832 | 0.298394 | 0.311922 | 0.674196 | 0.628244 |
| | DC | 1.15078 | 1.163144 | 1.205087 | 0.172039 | 0.17662 | 0.181954 | 0.995052 | 1.007908 | 1.025386 | 0.182627 | 0.192801 | 0.203502 | 0.754773 | 0.756169 |
| | mDC | 1.203537 | 1.223084 | 1.260971 | 0.16431 | 0.17307 | 0.177125 | 0.964833 | 0.985981 | 1.000818 | 0.195914 | 0.207708 | 0.218073 | 0.581056 | 0.630173 |
| | pDC | 1.286047 | 1.298494 | 1.324639 | 0.220003 | 0.224196 | 0.227362 | 1.095041 | 1.11744 | 1.155067 | 0.230754 | 0.248858 | 0.24918 | 1.013532 | 1.070474 |
| | NK | 1.313052 | 1.29091 | 1.347468 | 0.234238 | 0.234358 | 0.240017 | 1.334419 | 1.322469 | 1.326481 | 0.253424 | 0.253708 | 0.267181 | 0.797196 | 0.773671 |
| | CD16$^-$NK | 1.305995 | 1.276796 | 1.336781 | 0.248143 | 0.247455 | 0.253119 | 1.301771 | 1.306926 | 1.280568 | 0.264745 | 0.267535 | 0.277334 | 0.888156 | 0.848293 |
| | CD16$^+$NK | 1.393868 | 1.343438 | 1.429679 | 0.241979 | 0.250413 | 0.254401 | 1.443173 | 1.478379 | 1.473749 | 0.281466 | 0.278689 | 0.298215 | 0.651506 | 0.645168 |
| Adaptive | CD3$^+$T | 1.286254 | 1.214044 | 1.282096 | 0.555189 | 0.545772 | 0.554604 | 1.472075 | 1.458666 | 1.475067 | 0.181857 | 0.169591 | 0.186496 | 0.357149 | 0.335182 |
| | CD4$^+$T | 1.330136 | 1.245942 | 1.31962 | 0.514426 | 0.469789 | 0.503331 | 0.840896 | 0.8336 | 0.849089 | 0.138746 | 0.122949 | 0.138833 | 0.250576 | 0.229423 |
| | CD4$^+$T$_{EMRA}$ | 1.472357 | 1.235134 | 1.434 | 0.54189 | 0.443897 | 0.51765 | 1.229058 | 1.017168 | 1.135378 | 0.230152 | 0.155711 | 0.21135 | 0.359631 | 0.280359 |
| | CD4$^+$T$_{CM}$ | 1.520542 | 1.557475 | 1.502127 | 0.510449 | 0.545834 | 0.49587 | 0.920795 | 0.921958 | 0.900729 | 0.209338 | 0.217079 | 0.186671 | 0.360242 | 0.357255 |
| | CD4$^+$T$_{EM}$ | 1.285391 | 1.216198 | 1.281368 | 0.542881 | 0.485394 | 0.521246 | 0.793288 | 0.77919 | 0.805165 | 0.125363 | 0.113152 | 0.127382 | 0.229958 | 0.204343 |
| | CD4$^+$T$_{N}$ | 1.607182 | 1.613502 | 1.585248 | 0.480793 | 0.478444 | 0.482089 | 1.170465 | 1.100095 | 1.111578 | 0.26019 | 0.274959 | 0.263008 | 0.530275 | 0.476792 |
| | CD4$^+$T$_{Act}$ | 1.90856 | 1.816416 | 1.908364 | 0.927245 | 0.924228 | 0.950366 | 1.359995 | 1.255287 | 1.330313 | 0.381244 | 0.352956 | 0.390072 | 0.834793 | 0.773059 |
| | CD8$^+$T | 1.289709 | 1.234135 | 1.279933 | 0.732605 | 0.711399 | 0.717893 | 2.363867 | 2.369733 | 2.409506 | 0.195193 | 0.187689 | 0.19692 | 0.37497 | 0.362501 |
| | CD8$^+$T$_{EMRA}$ | 1.28221 | 1.197529 | 1.269731 | 0.570519 | 0.535898 | 0.561756 | 3.166549 | 3.074886 | 3.20788 | 0.206757 | 0.196153 | 0.208624 | 0.233429 | 0.216732 |
| | CD8$^+$T$_{CM}$ | 1.583797 | 1.580962 | 1.644775 | 1.046821 | 1.089686 | 1.057632 | 1.518407 | 1.275774 | 1.325354 | 0.286169 | 0.302176 | 0.301805 | 0.64726 | 0.690419 |
| | CD8$^+$T$_{EM}$ | 1.227502 | 1.180472 | 1.229313 | 0.917919 | 0.936702 | 0.934642 | 1.681301 | 1.587063 | 1.74406 | 0.164149 | 0.159422 | 0.169117 | 0.470087 | 0.466296 |
| | CD8$^+$T$_{N}$ | 1.44001 | 1.537044 | 1.447951 | 0.57504 | 0.610666 | 0.570772 | 2.225759 | 2.275759 | 2.116434 | 0.261695 | 0.334535 | 0.28508 | 0.331493 | 0.410877 |
| | CD8$^+$T$_{Act}$ | 1.537905 | 1.502801 | 1.54665 | 0.994546 | 1.013494 | 0.999666 | 2.051467 | 1.960427 | 1.933389 | 0.272991 | 0.273989 | 0.288873 | 0.640144 | 0.639561 |
| | Th1 | 1.603456 | 1.58657 | 1.612917 | 0.712098 | 0.729309 | 0.712065 | 0.946766 | 0.95923 | 0.955879 | 0.227674 | 0.234084 | 0.233081 | 0.439199 | 0.440876 |
| | Non-Th1/17 | 1.091171 | 1.047834 | 1.097662 | 0.373448 | 0.352008 | 0.370382 | 0.747891 | 0.756894 | 0.765964 | 0.083993 | 0.080028 | 0.088179 | 0.152694 | 0.147746 |
| | Th17 | 1.37712 | 1.333902 | 1.452683 | 0.428504 | 0.436787 | 0.430367 | 0.742835 | 0.91299 | 0.841801 | 0.126929 | 0.161784 | 0.164115 | 0.200492 | 0.270846 |
| | Treg | 2.412441 | 2.299336 | 2.387558 | 0.615347 | 0.591616 | 0.646843 | 1.116393 | 0.851604 | 1.05363 | 0.457026 | 0.410586 | 0.451505 | 0.996251 | 0.579849 |
| | Treg$_{Act}$ | 2.925021 | 3.020456 | 3.0562 | 0.7962 | 0.847731 | 0.835641 | 1.815221 | 1.836014 | 1.896151 | 0.856196 | 0.858272 | 0.919155 | 1.716503 | 1.433023 |
| | Treg$_{N}$ | 2.450785 | 2.390709 | 2.487156 | 0.573408 | 0.580318 | 0.589876 | 1.296116 | 1.129481 | 1.230502 | 0.531835 | 0.47997 | 0.53101 | 1.277689 | 0.872885 |
| | Treg$_{M}$ | 2.344148 | 2.220131 | 2.359672 | 0.813618 | 0.610248 | 0.837762 | 0.796756 | 0.748179 | 0.979746 | 0.399267 | 0.32207 | 0.391479 | 0.643572 | 0.474679 |
| | CD4$^+$CD8$^+$T | 2.275103 | 2.194975 | 2.237993 | 0.937597 | 1.014479 | 1.037743 | 2.54518 | 2.602163 | 2.626199 | 0.551424 | 0.612339 | 0.644994 | 0.803222 | 0.79375 |
| | GammaDeltaT | 1.826987 | 1.7653 | 1.765695 | 0.552676 | 0.50745 | 0.542736 | 2.316663 | 2.101113 | 2.274142 | 0.451835 | 0.415021 | 0.442937 | 1.001528 | 0.943049 |
| | B | 1.458161 | 1.416047 | 1.455894 | 0.280679 | 0.280434 | 0.282446 | 1.25547 | 1.285073 | 1.255187 | 0.321788 | 0.316736 | 0.327132 | 0.943482 | 0.935818 |
| | B$_{N}$ | 1.381911 | 1.344503 | 1.378723 | 0.266633 | 0.268173 | 0.270081 | 1.216225 | 1.250717 | 1.210659 | 0.301747 | 0.297661 | 0.306977 | 0.80267 | 0.793035 |
| | B$_{Mem}$ | 1.834096 | 1.80296 | 1.832589 | 0.354872 | 0.356374 | 0.356427 | 1.480848 | 1.484223 | 1.481229 | 0.460712 | 0.458248 | 0.462072 | 1.472383 | 1.464983 |
| | B$_{Trans}$ | 1.89175 | 1.854711 | 1.956919 | 0.370539 | 0.355563 | 0.37456 | 1.530133 | 1.46184 | 1.489519 | 0.452986 | 0.45086 | 0.473022 | 1.903623 | 1.953198 |
| | B$_{Pb}$ | 1.873418 | 1.844691 | 1.872951 | 0.357021 | 0.362627 | 0.358787 | 1.498115 | 1.513771 | 1.527042 | 0.470245 | 0.472044 | 0.472411 | 1.586039 | 1.580542 |

DENV vs Mock $p<0.05$

ZIKV vs Mock $P<0.05$

Zhao et al, Table S2

| | IFNg | | | IL6 | | | MIP1b | | | Perforin | | | TNFa | | |
|---|---|---|---|---|---|---|---|---|---|---|---|---|---|---|---|
| ZIKV | Mock | DENV | ZIKV | Mock | DENV | ZIKV | Mock | DENV | ZIKV | Mock | DENV | ZIKV | Mock | DENV | ZIKV |
| 0.689803 | 0.465325 | 0.456989 | 0.479693 | 0.342239 | 0.32819 | 0.357407 | 0.352815 | 0.349087 | 0.376103 | 0.413852 | 0.399259 | 0.4183 | 0.411405 | 0.400573 | 0.424306 |
| 0.707603 | 0.518424 | 0.530531 | 0.529182 | 0.379877 | 0.387126 | 0.392157 | 0.386667 | 0.409445 | 0.413208 | 0.464967 | 0.465727 | 0.466139 | 0.500334 | 0.522621 | 0.517203 |
| 0.694607 | 0.462466 | 0.451804 | 0.478266 | 0.340341 | 0.323075 | 0.356893 | 0.354865 | 0.347608 | 0.380737 | 0.418265 | 0.393701 | 0.420262 | 0.415338 | 0.389072 | 0.426833 |
| 0.791444 | 0.315033 | 0.327994 | 0.348131 | 0.205018 | 0.214363 | 0.235732 | 0.187955 | 0.203728 | 0.224199 | 0.365564 | 0.369978 | 0.386837 | 0.41112 | 0.42346 | 0.435662 |
| 0.655339 | 0.329753 | 0.348679 | 0.365622 | 0.217941 | 0.233676 | 0.25143 | 0.206508 | 0.225471 | 0.243173 | 0.340316 | 0.355774 | 0.365581 | 0.342502 | 0.360902 | 0.370999 |
| 1.068023 | 0.40119 | 0.411526 | 0.412531 | 0.281419 | 0.282886 | 0.295355 | 0.285099 | 0.297016 | 0.30675 | 0.443937 | 0.467646 | 0.463685 | 0.589253 | 0.608719 | 0.612667 |
| 0.831764 | 0.393866 | 0.397606 | 0.420633 | 0.274439 | 0.27271 | 0.298385 | 0.256715 | 0.259058 | 0.285013 | 0.567705 | 0.540063 | 0.55765 | 0.527719 | 0.520543 | 0.543063 |
| 0.911734 | 0.395594 | 0.394723 | 0.420394 | 0.277705 | 0.272467 | 0.299425 | 0.262986 | 0.261795 | 0.290651 | 0.581915 | 0.568978 | 0.568795 | 0.56248 | 0.560685 | 0.577484 |
| 0.702404 | 0.425472 | 0.439616 | 0.452697 | 0.29172 | 0.298495 | 0.316512 | 0.268151 | 0.278052 | 0.30062 | 0.548218 | 0.537662 | 0.547448 | 0.559716 | 0.574676 | 0.59474 |
| 0.370177 | 0.411269 | 0.389128 | 0.414112 | 0.266396 | 0.243347 | 0.271313 | 0.255689 | 0.233384 | 0.262062 | 0.480287 | 0.456304 | 0.483522 | 0.456877 | 0.440206 | 0.469261 |
| 0.24869 | 0.42695 | 0.395365 | 0.427168 | 0.29923 | 0.2686 | 0.300958 | 0.250747 | 0.223874 | 0.252212 | 0.32774 | 0.296163 | 0.326158 | 0.338424 | 0.320786 | 0.345566 |
| 0.349428 | 0.512711 | 0.424714 | 0.501254 | 0.374377 | 0.294215 | 0.373068 | 0.336604 | 0.253861 | 0.323404 | 0.480601 | 0.369319 | 0.467626 | 0.506458 | 0.45122 | 0.519033 |
| 0.337197 | 0.506891 | 0.554148 | 0.510108 | 0.372616 | 0.409122 | 0.374038 | 0.340659 | 0.383352 | 0.339735 | 0.391484 | 0.423847 | 0.385025 | 0.437761 | 0.471475 | 0.433602 |
| 0.228894 | 0.400245 | 0.375318 | 0.405271 | 0.279037 | 0.253579 | 0.282498 | 0.227862 | 0.207698 | 0.235178 | 0.297255 | 0.273247 | 0.302 | 0.283104 | 0.261506 | 0.28996 |
| 0.444186 | 0.57519 | 0.609004 | 0.566517 | 0.462088 | 0.457525 | 0.461845 | 0.435581 | 0.44331 | 0.423673 | 0.528156 | 0.505801 | 0.515575 | 0.744898 | 0.707226 | 0.728119 |
| 0.830429 | 0.738213 | 0.697283 | 0.740431 | 0.598668 | 0.551895 | 0.602218 | 0.595249 | 0.552104 | 0.600524 | 0.68422 | 0.63239 | 0.680337 | 0.681885 | 0.626603 | 0.683347 |
| 0.376487 | 0.411376 | 0.396639 | 0.411105 | 0.25375 | 0.240658 | 0.255299 | 0.258361 | 0.244444 | 0.259377 | 0.557165 | 0.540663 | 0.560809 | 0.539603 | 0.539976 | 0.546481 |
| 0.24055 | 0.406415 | 0.385162 | 0.408485 | 0.241323 | 0.225272 | 0.245106 | 0.240386 | 0.221064 | 0.241819 | 0.644278 | 0.608631 | 0.649781 | 0.683212 | 0.70049 | 0.694028 |
| 0.700689 | 0.555948 | 0.562905 | 0.581607 | 0.406868 | 0.42089 | 0.43957 | 0.4098 | 0.418992 | 0.433856 | 0.581545 | 0.547435 | 0.586996 | 0.484 | 0.478852 | 0.51236 |
| 0.475427 | 0.389815 | 0.380364 | 0.397768 | 0.239189 | 0.231668 | 0.248396 | 0.242079 | 0.234996 | 0.251123 | 0.48235 | 0.464146 | 0.487949 | 0.351261 | 0.33925 | 0.364379 |
| 0.324982 | 0.500689 | 0.538332 | 0.483991 | 0.344671 | 0.406243 | 0.371659 | 0.342404 | 0.402878 | 0.343234 | 0.606191 | 0.640671 | 0.602271 | 0.840139 | 0.953286 | 0.898605 |
| 0.661216 | 0.524095 | 0.523589 | 0.537106 | 0.371137 | 0.36904 | 0.387066 | 0.378092 | 0.380436 | 0.395842 | 0.613685 | 0.605942 | 0.626531 | 0.519928 | 0.517527 | 0.531097 |
| 0.456621 | 0.549629 | 0.551359 | 0.556668 | 0.413383 | 0.413115 | 0.422446 | 0.370829 | 0.373607 | 0.379842 | 0.448955 | 0.451012 | 0.458785 | 0.421746 | 0.425938 | 0.436372 |
| 0.157754 | 0.327438 | 0.312 | 0.335374 | 0.209445 | 0.192839 | 0.2169 | 0.164431 | 0.1509 | 0.172857 | 0.23851 | 0.22736 | 0.244505 | 0.282416 | 0.272898 | 0.291881 |
| 0.23386 | 0.419701 | 0.42821 | 0.462057 | 0.318111 | 0.308983 | 0.346002 | 0.241839 | 0.263208 | 0.290993 | 0.288473 | 0.333256 | 0.32695 | 0.301688 | 0.386444 | 0.372939 |
| 0.816467 | 0.901542 | 0.806316 | 0.901736 | 0.81929 | 0.739141 | 0.80227 | 0.8042 | 0.675 | 0.795475 | 0.735279 | 0.585585 | 0.694177 | 0.900286 | 0.708275 | 0.860227 |
| 1.602836 | 1.184426 | 1.251537 | 1.297832 | 1.121765 | 1.158177 | 1.211696 | 1.191105 | 1.264822 | 1.332099 | 1.106175 | 1.063862 | 1.155917 | 1.500866 | 1.393741 | 1.466943 |
| 1.178747 | 0.94361 | 0.878823 | 0.986849 | 0.874965 | 0.82518 | 0.902855 | 0.893305 | 0.797164 | 0.92829 | 0.834253 | 0.702237 | 0.826576 | 1.109124 | 0.917043 | 1.109187 |
| 0.568251 | 0.813531 | 0.754265 | 0.819548 | 0.715018 | 0.674821 | 0.725034 | 0.665034 | 0.624582 | 0.658303 | 0.589218 | 0.507765 | 0.606141 | 0.613709 | 0.488905 | 0.635277 |
| 0.793768 | 0.964364 | 0.976433 | 0.985943 | 0.788254 | 0.835397 | 0.850581 | 0.817869 | 0.853649 | 0.885694 | 1.03643 | 1.057288 | 1.073303 | 1.235776 | 1.280254 | 1.318013 |
| 0.984259 | 0.736874 | 0.70113 | 0.707389 | 0.569372 | 0.551009 | 0.553333 | 0.626018 | 0.59398 | 0.595037 | 0.823746 | 0.819759 | 0.848632 | 0.962512 | 0.943058 | 0.928486 |
| 0.947454 | 0.453719 | 0.44827 | 0.458705 | 0.335602 | 0.323665 | 0.343954 | 0.34459 | 0.342078 | 0.356454 | 0.542442 | 0.541292 | 0.547111 | 0.881099 | 0.89423 | 0.87641 |
| 0.813397 | 0.414164 | 0.410448 | 0.419151 | 0.298025 | 0.289101 | 0.306808 | 0.303334 | 0.303095 | 0.314322 | 0.506699 | 0.509119 | 0.509358 | 0.840973 | 0.857636 | 0.83789 |
| 1.511121 | 0.65655 | 0.657462 | 0.662952 | 0.53378 | 0.527978 | 0.541143 | 0.553825 | 0.561601 | 0.56698 | 0.720705 | 0.717855 | 0.726091 | 1.093644 | 1.123136 | 1.088121 |
| 2.046221 | 0.654244 | 0.64246 | 0.685239 | 0.53961 | 0.515789 | 0.572326 | 0.566242 | 0.545794 | 0.608048 | 0.780895 | 0.74999 | 0.799719 | 1.09931 | 1.07603 | 1.091687 |
| 1.600393 | 0.674659 | 0.678218 | 0.680076 | 0.552116 | 0.550572 | 0.562218 | 0.577661 | 0.588156 | 0.589571 | 0.744729 | 0.748828 | 0.753123 | 1.090447 | 1.115883 | 1.086734 |
