# Supplementary material for: Single cell immune profiling of dengue virus patients reveals intact immune responses to Zika virus with enrichment of innate immune signatures
Source: PLoS Negl Trop Dis. 2020 Mar 9;14(3):e0008112. doi: 10.1371/journal.pntd.0008112 (PMC7082063; doi:10.1371/journal.pntd.0008112)
Supplement: S3 Table — Columns show p value for differences vs mock for cell subset-activation marker combinations in response to infection with dengue or Zika virus in vitro. P values for dengue patients at acute and convalescent time points and well subjects are shown with differences p<0.05 highlighted in orange. (PDF) [file pntd.0008112.s009.pdf]

Table S3 Ranked sum of p values for enrichment of cell/activation markers

			Acute-Dengue	Acute-Zika	Conv-Dengue	Conv-Zika	Well-Dengue	Well-Zika
is_innate	cell	cyto	p_Stim	p_Stim	p_Stim	p_Stim	p_Stim	p_Stim
FALSE	CD4+ TAct	CD152	0.02300873	0.995384394	0.268541863	0.566225079	0.21742629	0.043389145
FALSE	CD4+ TAct	CD279	0.914935883	0.256149166	0.650630018	0.835754515	0.626536419	0.527090443
FALSE	CD4+ TAct	CD57	0.04357313	0.480872472	0.442023059	0.960763413	0.221427186	0.988497622
FALSE	CD4+ TAct	CD69	0.021537285	0.516350612	0.914206954	0.882258851	0.642548042	0.123284504
FALSE	CD4+ TAct	IFNb	0.005230455	0.833112129	0.614619982	0.7823237	0.217405938	0.362698215
FALSE	CD4+ TAct	IFNg	0.001566773	0.861445469	0.302505326	0.947509394	0.094410492	0.021123407
FALSE	CD4+ TAct	IL6	0.00114	0.808239494	0.250332478	0.899764487	0.060544234	0.075968561
FALSE	CD4+ TAct	MIP1b	0.005720245	0.694323263	0.513332012	0.893268889	0.162242903	0.012430509
FALSE	CD4+ TAct	Perforin	0.002411181	0.794358686	0.363010279	0.951072034	0.032173664	0.697237221
FALSE	CD4+ TAct	TNFa	0.034380062	0.944597956	0.722932169	0.81941191	0.352345559	0.21970702
FALSE	CD8+ TAct	CD152	0.26616297	0.776704007	0.454485373	0.375378134	0.499615703	0.057622422
FALSE	CD8+ TAct	CD279	0.277048755	0.71797931	0.818001928	0.72861109	0.324538809	0.68893109
FALSE	CD8+ TAct	CD57	0.235239111	0.22907784	0.861317359	0.506334372	0.869225142	0.790270319
FALSE	CD8+ TAct	CD69	0.86262858	0.101755006	0.761639594	0.919800845	0.922568008	0.02802925
FALSE	CD8+ TAct	IFNb	0.941703418	0.064645813	0.941866572	0.98862698	0.87156732	0.007238389
FALSE	CD8+ TAct	IFNg	0.942487824	0.171552701	0.699248749	0.685676452	0.920524175	0.003486552
FALSE	CD8+ TAct	IL6	0.822686266	0.159228324	0.624476906	0.598934577	0.874776315	0.006367336
FALSE	CD8+ TAct	MIP1b	0.804911245	0.082700021	0.868588222	0.787783653	0.951740729	0.00655001
FALSE	CD8+ TAct	Perforin	0.443524235	0.422901779	0.835017242	0.612746926	0.916658396	0.034156054
FALSE	CD8+ TAct	TNFa	0.839662699	0.503738506	0.987381884	0.730331012	0.640263758	0.043308649
FALSE	TregAct	CD152	0.422375226	0.20102858	0.758437022	0.168115803	0.467441621	0.123603428
FALSE	TregAct	CD279	0.432592717	0.535210353	0.419989118	0.574050183	0.911681739	0.424196514
FALSE	TregAct	CD57	0.854189335	0.501000438	0.93418426	0.215435906	0.299107577	0.282257267
FALSE	TregAct	CD69	0.93679155	0.301046065	0.656175136	0.394847852	0.524044693	0.075116751
FALSE	TregAct	IFNb	0.144196707	0.209168814	0.076220815	0.444113407	0.481501415	0.280124452
FALSE	TregAct	IFNg	0.463846561	0.144820426	0.652729139	0.231328895	0.932984281	0.326551225
FALSE	TregAct	IL6	0.658759372	0.178482681	0.940543918	0.408985832	0.972834789	0.148634147
FALSE	TregAct	MIP1b	0.330461404	0.041861014	0.626350608	0.140642166	0.510564959	0.249412443
FALSE	TregAct	Perforin	0.457366754	0.512544936	0.39057006	0.899623793	0.469356906	0.358590835
FALSE	TregAct	TNFa	0.190245389	0.654611115	0.393934514	0.922272348	0.657256279	0.69288801
FALSE	B	CD152	0.221354955	0.941008582	0.251715763	0.384280077	0.371561952	0.428674066
FALSE	B	CD279	0.991367315	0.590370543	0.77445045	0.776393263	0.35780531	0.766007805
FALSE	B	CD57	0.128323772	0.983091485	0.387966943	0.634847768	0.435850865	0.863244576
FALSE	B	CD69	0.739113539	0.390490672	0.540482602	0.94671055	0.808180793	0.84591937
FALSE	B	IFNb	0.675128192	0.889234345	0.92852595	0.717754502	0.360352186	0.03168478
FALSE	B	IFNg	0.531078223	0.479226303	0.716846239	0.974634243	0.399673807	0.046193299
FALSE	B	IL6	0.186394324	0.340269672	0.466702616	0.953898812	0.844865342	0.058676381
FALSE	B	MIP1b	0.816776738	0.139383313	0.79072975	0.920768738	0.926269258	0.038244545
FALSE	B	Perforin	0.868830052	0.608311584	0.829905457	0.907001678	0.825616977	0.06424518
FALSE	B	TNFa	0.332182217	0.674051421	0.969469211	0.562809824	0.599849983	0.828789333
TRUE	CD16-NK	CD152	0.30768959	0.395841468	0.438698124	0.949722286	0.53385732	0.083921429
TRUE	CD16-NK	CD279	0.811904079	0.262162861	0.929414313	0.66190467	0.7043019	0.17693193
TRUE	CD16-NK	CD57	0.85471865	0.375136944	0.941617276	0.314521308	0.127825288	0.225478492
TRUE	CD16-NK	CD69	0.821426499	0.19476378	0.944407629	0.662507077	0.459668144	0.025232122
TRUE	CD16-NK	IFNb	0.092076954	0.317421977	0.86815009	0.665359891	0.445153238	0.24612046
TRUE	CD16-NK	IFNg	0.789585417	0.016954104	0.634642555	0.458206161	0.839147628	0.00591157
TRUE	CD16-NK	IL6	0.424539135	0.071589746	0.698039122	0.581297666	0.921416481	0.058378825
TRUE	CD16-NK	MIP1b	0.776095147	0.019886939	0.867394477	0.429901268	0.629624233	0.012727578
TRUE	CD16-NK	Perforin	0.606226988	0.697633099	0.967578174	0.841320682	0.598316469	0.87461166
TRUE	CD16-NK	TNFa	0.796263501	0.175636736	0.827968867	0.897372363	0.54745203	0.070209574
TRUE	CD16+NK	CD152	0.127379258	0.317118765	0.563797405	0.960335261	0.820717535	0.143811075
TRUE	CD16+NK	CD279	0.45772303	0.066620534	0.59180011	0.407099305	0.180925242	0.104599863
TRUE	CD16+NK	CD57	0.514554841	0.204759379	0.855463636	0.967351255	0.337225939	0.146326407
TRUE	CD16+NK	CD69	0.710496777	0.119395846	0.687093992	0.599686285	0.551107825	0.109549326
TRUE	CD16+NK	IFNb	0.756807624	0.037905107	0.855686915	0.639615265	0.10628377	0.005005304
TRUE	CD16+NK	IFNg	0.277172384	0.017660592	0.680289408	0.505944318	0.059059349	0.016437298
TRUE	CD16+NK	IL6	0.6328561	0.087089979	0.760665266	0.780390221	0.142618013	0.053145817
TRUE	CD16+NK	MIP1b	0.281376394	0.011783721	0.585156043	0.414185492	0.072721966	0.026478017

Zhao et al Table S3

TRUE	CD16+NK	Perforin	0.735412245	0.981367113	0.931600666	0.758842043	0.685762545	0.315203813
TRUE	CD16+NK	TNFa	0.444084672	0.019655821	0.776517008	0.828308204	0.093587418	0.01015982
FALSE	CD3+T	CD152	0.007232988	0.867297907	0.13081841	0.458102256	0.035784986	0.139695142
FALSE	CD3+T	CD279	0.283497453	0.938879044	0.488021895	0.937923579	0.578237316	0.348080944
FALSE	CD3+T	CD57	0.715038842	0.894317776	0.905742188	0.721377804	0.585581808	0.802013522
FALSE	CD3+T	CD69	0.004637415	0.427332862	0.516604201	0.931609133	0.244778142	0.24893819
FALSE	CD3+T	IFNb	0.015722855	0.283968113	0.600943977	0.960004143	0.24678665	0.108384291
FALSE	CD3+T	IFNg	0.002843975	0.657605803	0.210404172	0.876317525	0.09028764	0.020255175
FALSE	CD3+T	IL6	0.001926768	0.501139876	0.180331244	0.811821848	0.113838559	0.047351937
FALSE	CD3+T	MIP1b	0.00462921	0.382282728	0.26990414	0.920608176	0.156192369	0.048099428
FALSE	CD3+T	Perforin	0.031521213	0.665278147	0.21542222	0.895281212	0.163658653	0.064097153
FALSE	CD3+T	TNFa	0.117499837	0.067987114	0.376406249	0.894426236	0.590448123	0.05311577
FALSE	CD4+CD8+T	CD152	0.737876442	0.884767816	0.552699446	0.658061528	0.98742646	0.227718427
FALSE	CD4+CD8+T	CD279	0.500738502	0.117537221	0.39653781	0.616836498	0.735298141	0.039267175
FALSE	CD4+CD8+T	CD57	0.92634778	0.843135954	0.213412414	0.919053895	0.572545577	0.449695356
FALSE	CD4+CD8+T	CD69	0.680776806	0.575381085	0.914825968	0.718586374	0.668540948	0.230662822
FALSE	CD4+CD8+T	IFNb	0.895927676	0.942426944	0.56515872	0.330290004	0.93129972	0.367342226
FALSE	CD4+CD8+T	IFNg	0.968569689	0.899311542	0.128758699	0.363964645	0.62936722	0.263125437
FALSE	CD4+CD8+T	IL6	0.803080629	0.672684004	0.188773044	0.53298449	0.656475658	0.266733774
FALSE	CD4+CD8+T	MIP1b	0.890587756	0.756167705	0.312574792	0.392307917	0.611949273	0.240596109
FALSE	CD4+CD8+T	Perforin	0.93473945	0.849618262	0.080199409	0.152076578	0.712353794	0.350129132
FALSE	CD4+CD8+T	TNFa	0.887921184	0.769109763	0.792520712	0.487254368	0.752572647	0.374885615
FALSE	CD4+T	CD152	0.004142241	0.668116528	0.09626653	0.426444793	0.026646588	0.151224883
FALSE	CD4+T	CD279	0.00192323	0.21654653	0.336408621	0.953364503	0.106072121	0.742909941
FALSE	CD4+T	CD57	0.531509612	0.318277558	0.938203631	0.824668095	0.949301354	0.440726212
FALSE	CD4+T	CD69	0.008802634	0.983802076	0.386933142	0.944201377	0.310853526	0.239494334
FALSE	CD4+T	IFNb	0.018655674	0.833811792	0.642789917	0.873026766	0.136553884	0.218506452
FALSE	CD4+T	IFNg	0.000627669	0.972310473	0.096817496	0.819588405	0.018566855	0.03020083
FALSE	CD4+T	IL6	0.000236941	0.79125382	0.066151839	0.724605026	0.022937681	0.075959852
FALSE	CD4+T	MIP1b	0.001392821	0.795301174	0.173939994	0.883145344	0.02466513	0.095134057
FALSE	CD4+T	Perforin	0.001717274	0.794980563	0.129558894	0.828918106	0.027188037	0.04373752
FALSE	CD4+T	TNFa	0.012832168	0.145780999	0.21335138	0.934889739	0.197940707	0.028350605
FALSE	CD8+T	CD152	0.033765761	0.668969654	0.243597307	0.543388978	0.066523383	0.398236472
FALSE	CD8+T	CD279	0.03386533	0.275027746	0.608207567	0.929694209	0.144632658	0.627918953
FALSE	CD8+T	CD57	0.800418211	0.147540051	0.984306492	0.725010873	0.874866533	0.26575745
FALSE	CD8+T	CD69	0.101751755	0.712581324	0.668961521	0.968657135	0.343640818	0.265733667
FALSE	CD8+T	IFNb	0.036816259	0.863378232	0.788136	0.880871156	0.207862641	0.243771805
FALSE	CD8+T	IFNg	0.045169774	0.970587209	0.363324174	0.837291873	0.201568257	0.076470711
FALSE	CD8+T	IL6	0.043396462	0.826075327	0.36502478	0.878872092	0.320299228	0.099349582
FALSE	CD8+T	MIP1b	0.051575504	0.885162589	0.444928037	0.879735959	0.272464714	0.166623973
FALSE	CD8+T	Perforin	0.09724511	0.722261653	0.338788144	0.876076407	0.293526579	0.089722714
FALSE	CD8+T	TNFa	0.975120628	0.441467308	0.899269122	0.98173146	0.939436563	0.144797937
FALSE	CD4+TCM	CD152	0.465839674	0.747701486	0.977049611	0.069002892	0.005379825	0.0123853
FALSE	CD4+TCM	CD279	0.29144771	0.625660998	0.625381069	0.795622905	0.00970973	0.586599356
FALSE	CD4+TCM	CD57	0.990252227	0.534263576	0.847257413	0.409146987	0.753953567	0.760986164
FALSE	CD4+TCM	CD69	0.555823408	0.437113729	0.900768566	0.211015381	0.044027507	0.210107014
FALSE	CD4+TCM	IFNb	0.948714796	0.560519458	0.934472717	0.172128023	0.152786266	0.118197288
FALSE	CD4+TCM	IFNg	0.114167077	0.90950088	0.405308461	0.205218607	0.00107859	0.00293122
FALSE	CD4+TCM	IL6	0.174670512	0.953005352	0.480658961	0.240929393	0.001060056	0.008742641
FALSE	CD4+TCM	MIP1b	0.15233024	0.976207487	0.542455274	0.205149048	0.00135055	0.009820978
FALSE	CD4+TCM	Perforin	0.293925302	0.80944692	0.788451651	0.23127356	0.002329544	0.006701657
FALSE	CD4+TCM	TNFa	0.151130006	0.906668124	0.752941356	0.217579656	0.000870799	0.000599385
FALSE	CD8+TCM	CD152	0.992793325	0.411335134	0.460630597	0.645778862	0.358574387	0.184707093
FALSE	CD8+TCM	CD279	0.394908248	0.803177538	0.459566738	0.696325506	0.578633823	0.911060925
FALSE	CD8+TCM	CD57	0.046461766	0.101850687	0.44164949	0.338451745	0.178998684	0.499340392
FALSE	CD8+TCM	CD69	0.508774588	0.595475029	0.829642457	0.839719021	0.186208601	0.305583521
FALSE	CD8+TCM	IFNb	0.22451175	0.211354456	0.906814258	0.783392808	0.161315408	0.129679709
FALSE	CD8+TCM	IFNg	0.759972447	0.290154874	0.984120673	0.861452305	0.566378132	0.141364405
FALSE	CD8+TCM	IL6	0.566303357	0.293620205	0.924938314	0.808184135	0.18057749	0.140599528
FALSE	CD8+TCM	MIP1b	0.689061666	0.408931003	0.915392818	0.865422211	0.212607618	0.120355966
FALSE	CD8+TCM	Perforin	0.359692702	0.878136569	0.267565416	0.854077226	0.894480607	0.514415707

FALSE	CD8+TCM	TNFa	0.912723974	0.496917391	0.702021262	0.926458285	0.426972055	0.263392061
TRUE	CD16-Mono	CD152	0.669414771	0.905629911	0.621626066	0.561580876	0.698239569	0.865707171
TRUE	CD16-Mono	CD279	0.192308569	0.311046845	0.726475516	0.831667015	0.530058235	0.820875549
TRUE	CD16-Mono	CD57	0.439503045	0.969421365	0.578352339	0.637785135	0.169994932	0.096056923
TRUE	CD16-Mono	CD69	0.791780483	0.645314018	0.96455848	0.97515492	0.139493979	0.506606733
TRUE	CD16-Mono	IFNb	0.474078825	0.596934508	0.665896474	0.663218789	0.218880859	0.231318663
TRUE	CD16-Mono	IFNg	0.602408217	0.607328139	0.698879248	0.963428681	0.557317546	0.768230275
TRUE	CD16-Mono	IL6	0.782333095	0.536157724	0.697254088	0.80255239	0.927624534	0.943480935
TRUE	CD16-Mono	MIP1b	0.289545413	0.214166619	0.93693549	0.926138399	0.93288079	0.982686589
TRUE	CD16-Mono	Perforin	0.975872068	0.920743588	0.506285869	0.768751986	0.277943218	0.073037741
TRUE	CD16-Mono	TNFa	0.205746715	0.173724748	0.744028746	0.760275084	0.000916384	0.01298794
TRUE	DC	CD152	0.685453676	0.064840131	0.823210842	0.675362319	0.740375008	0.001938026
TRUE	DC	CD279	0.350540206	0.004435217	0.238670026	0.117427112	0.366827447	0.055557067
TRUE	DC	CD57	0.126961906	0.001550288	0.656160682	0.505329609	0.741629729	0.046593395
TRUE	DC	CD69	0.143650308	0.001577839	0.40427188	0.2329647	0.363013783	0.007477413
TRUE	DC	IFNb	0.978218766	0.080374775	0.80192765	0.575621525	0.856141028	0.176466802
TRUE	DC	IFNg	0.071845284	2.88E-05	0.578866294	0.121584371	0.208934678	1.19E-05
TRUE	DC	IL6	0.192615187	0.000359273	0.715076532	0.256653713	0.210663964	0.001470669
TRUE	DC	MIP1b	0.056098579	7.85E-05	0.363425988	0.152003044	0.063718938	6.45E-05
TRUE	DC	Perforin	0.550804195	0.012954462	0.994476238	0.614147455	0.310866439	0.007295943
TRUE	DC	TNFa	0.294304707	0.019285405	0.96922631	0.731415441	0.527422986	0.023490427
FALSE	CD4+TEMRA	CD152	0.000198725	0.31530546	0.01087307	0.44874427	0.003158841	0.686667632
FALSE	CD4+TEMRA	CD279	0.001816258	0.131676155	0.023694212	0.959583158	0.013604682	0.504502588
FALSE	CD4+TEMRA	CD57	0.118250072	0.460228523	0.235975762	0.424316054	0.246554581	0.284602893
FALSE	CD4+TEMRA	CD69	0.000379819	0.174208268	0.105290118	0.949620571	0.015541576	0.259489323
FALSE	CD4+TEMRA	IFNb	0.000776128	0.535255357	0.291051645	0.79483713	0.024639478	0.568903611
FALSE	CD4+TEMRA	IFNg	0.000450288	0.316311463	0.017276323	0.831901464	0.013449482	0.532323938
FALSE	CD4+TEMRA	IL6	0.00034997	0.922594416	0.013014527	0.784217655	0.016936278	0.285983417
FALSE	CD4+TEMRA	MIP1b	0.000705435	0.241289433	0.087339486	0.849716258	0.008580095	0.776707067
FALSE	CD4+TEMRA	Perforin	0.000398051	0.394643649	0.032146869	0.986643885	0.074205698	0.77495163
FALSE	CD4+TEMRA	TNFa	0.055342348	0.433775197	0.299370879	0.833399064	0.359051964	0.220444882
FALSE	CD8+TEMRA	CD152	0.023960484	0.668063859	0.113124704	0.529687451	0.102945145	0.426010352
FALSE	CD8+TEMRA	CD279	0.00700663	0.358515056	0.22957751	0.987011036	0.137653613	0.633855109
FALSE	CD8+TEMRA	CD57	0.195926889	0.45464214	0.586536891	0.821525773	0.29202437	0.925759749
FALSE	CD8+TEMRA	CD69	0.114984284	0.739980831	0.519689781	0.930890893	0.301172096	0.636229032
FALSE	CD8+TEMRA	IFNb	0.076763247	0.325443921	0.158260165	0.904169254	0.675322938	0.010538002
FALSE	CD8+TEMRA	IFNg	0.0324687	0.800961342	0.239667422	0.893029409	0.23621931	0.070061044
FALSE	CD8+TEMRA	IL6	0.082731593	0.660807721	0.256048154	0.922611821	0.564123145	0.051488735
FALSE	CD8+TEMRA	MIP1b	0.042171578	0.858419242	0.312454579	0.92453124	0.37096441	0.102711587
FALSE	CD8+TEMRA	Perforin	0.019667225	0.694523557	0.139963869	0.814622894	0.177775387	0.245571539
FALSE	CD8+TEMRA	TNFa	0.33403086	0.329534193	0.910163303	0.929749592	0.450683386	0.234480823
FALSE	CD4+TEM	CD152	0.031686762	0.881562432	0.099287347	0.344686688	0.186571774	0.190958197
FALSE	CD4+TEM	CD279	0.002867872	0.201626521	0.33563824	0.867536981	0.147274052	0.768544975
FALSE	CD4+TEM	CD57	0.230652614	0.267737951	0.751662522	0.653734417	0.376327547	0.28759424
FALSE	CD4+TEM	CD69	0.023286786	0.656885224	0.39264471	0.874828326	0.789821694	0.124585253
FALSE	CD4+TEM	IFNb	0.002665549	0.908241545	0.536063755	0.89158634	0.09420815	0.391771573
FALSE	CD4+TEM	IFNg	0.012809228	0.480288348	0.07154179	0.746141222	0.291345753	0.015174031
FALSE	CD4+TEM	IL6	0.005710299	0.638786283	0.04435966	0.621299504	0.251341563	0.093857074
FALSE	CD4+TEM	MIP1b	0.019070207	0.225917834	0.111001032	0.90077995	0.362514268	0.088981502
FALSE	CD4+TEM	Perforin	0.013726043	0.488992889	0.086557433	0.880698805	0.377456279	0.004674453
FALSE	CD4+TEM	TNFa	0.037728279	0.306628301	0.096745503	0.923255468	0.959560409	0.003077342
FALSE	CD8+TEM	CD152	0.08204529	0.940307438	0.101883105	0.63562663	0.151773945	0.207712605
FALSE	CD8+TEM	CD279	0.285670806	0.301667978	0.663747511	0.826874723	0.25654966	0.223661125
FALSE	CD8+TEM	CD57	0.085351527	0.176191468	0.671411794	0.380550989	0.172604316	0.050534232
FALSE	CD8+TEM	CD69	0.237648003	0.251905197	0.622852531	0.786692829	0.472957156	0.149808914
FALSE	CD8+TEM	IFNb	0.516787572	0.453599228	0.809536805	0.927747299	0.85501007	0.493060005
FALSE	CD8+TEM	IFNg	0.154088603	0.256656136	0.241490065	0.892791819	0.198143921	0.077968267
FALSE	CD8+TEM	IL6	0.209316828	0.189906543	0.252850149	0.87830625	0.437401839	0.069367911
FALSE	CD8+TEM	MIP1b	0.245551957	0.170824309	0.385520131	0.87148381	0.477313949	0.121885976
FALSE	CD8+TEM	Perforin	0.031780057	0.514652274	0.111852429	0.527654041	0.166145962	0.160720139
FALSE	CD8+TEM	TNFa	0.042918673	0.053056223	0.351074616	0.714481078	0.387919563	0.022400083

Zhao et al Table S3

FALSE	γδT	CD152	0.203279411	0.151357692	0.450144883	0.486112699	0.634602477	0.821680902
FALSE	γδT	CD279	0.095960914	0.576034111	0.584516854	0.993219318	0.164059129	0.397942784
FALSE	γδT	CD57	0.075346553	0.682302981	0.842647981	0.512986153	0.007649925	0.48127444
FALSE	γδT	CD69	0.128047939	0.604687805	0.562589385	0.967881849	0.735691275	0.574848632
FALSE	γδT	IFNb	0.255163823	0.729679306	0.813423074	0.915536351	0.67151133	0.92181687
FALSE	γδT	IFNg	0.158842583	0.096415958	0.558022012	0.775043593	0.33767397	0.860326825
FALSE	γδT	IL6	0.558818044	0.395225495	0.665918523	0.724435246	0.535978366	0.725557409
FALSE	γδT	MIP1b	0.228109169	0.145130386	0.64891442	0.90999256	0.392231524	0.844530192
FALSE	γδT	Perforin	0.958483555	0.567367419	0.91388335	0.781424395	0.141255594	0.747853595
FALSE	γδT	TNFa	0.471931823	0.154363098	0.806576074	0.761976046	0.147588997	0.532354635
FALSE	BMem	CD152	0.390986339	0.962508189	0.241409834	0.549471902	0.920928593	0.057086728
FALSE	BMem	CD279	0.763187509	0.673774838	0.877199654	0.678523757	0.600971483	0.949473106
FALSE	BMem	CD57	0.893986229	0.990469796	0.792617135	0.420517477	0.689299229	0.413342676
FALSE	BMem	CD69	0.892083559	0.834016742	0.685292744	0.883153286	0.848128398	0.705941511
FALSE	BMem	IFNb	0.801524999	0.308605932	0.973807884	0.902450048	0.993807191	0.011901442
FALSE	BMem	IFNg	0.922535159	0.532866749	0.482708098	0.647005187	0.575464632	0.001092103
FALSE	BMem	IL6	0.591746756	0.506112998	0.281841158	0.752204955	0.383509628	0.003776592
FALSE	BMem	MIP1b	0.523918666	0.20855651	0.528372606	0.606147399	0.348767738	0.003536982
FALSE	BMem	Perforin	0.854668077	0.705337459	0.563736575	0.645029123	0.361345636	0.149456674
FALSE	BMem	TNFa	0.159808308	0.69884479	0.866366766	0.836061188	0.770515906	0.413434496
FALSE	TregMem	CD152	0.065945842	0.749549558	0.275307464	0.680959353	0.241207762	0.168311581
FALSE	TregMem	CD279	0.02006845	0.792147841	0.05991431	0.642509528	0.050710968	0.884208991
FALSE	TregMem	CD57	0.424667169	0.067475966	0.15309469	0.970958526	0.790130022	0.02528579
FALSE	TregMem	CD69	0.06333638	0.84834606	0.09424484	0.231364067	0.509057897	0.084243112
FALSE	TregMem	IFNb	0.02248369	0.227295968	0.077554199	0.226124308	0.12185308	0.929320507
FALSE	TregMem	IFNg	0.126381991	0.857029826	0.118862662	0.924185666	0.27464669	0.50405665
FALSE	TregMem	IL6	0.299747586	0.770518575	0.274814828	0.950892498	0.585822289	0.292844207
FALSE	TregMem	MIP1b	0.407644499	0.82905926	0.243398796	0.790859239	0.726301419	0.62274464
FALSE	TregMem	Perforin	0.131730048	0.72105439	0.039520562	0.721734824	0.408633034	0.313829589
FALSE	TregMem	TNFa	0.035777066	0.753585684	0.039499159	0.481885439	0.311513157	0.154867414
TRUE	Mono	CD152	0.565477531	0.276196473	0.579431272	0.976220708	0.081345207	0.424974125
TRUE	Mono	CD279	0.478970901	0.15771544	0.883900591	0.829580197	0.314972628	0.45925789
TRUE	Mono	CD57	0.496084082	0.928650046	0.985431809	0.888574047	0.024782806	0.376356246
TRUE	Mono	CD69	0.849069652	0.218900439	0.912073236	0.801953521	0.117950449	0.520591252
TRUE	Mono	IFNb	0.051307993	0.233753457	0.600426154	0.852087415	0.215853215	0.088112761
TRUE	Mono	IFNg	0.509248144	0.23775289	0.816877361	0.750574291	0.033440529	0.805150434
TRUE	Mono	IL6	0.25097736	0.222946491	0.69240131	0.975380788	0.030048703	0.686473816
TRUE	Mono	MIP1b	0.786724161	0.08067032	0.915171848	0.755973267	0.058715338	0.615823747
TRUE	Mono	Perforin	0.207145105	0.693207159	0.408506361	0.788997418	0.046149532	0.257658473
TRUE	Mono	TNFa	0.282482614	0.196340374	0.58756358	0.685753457	0.468599856	0.227124103
TRUE	mDC	CD152	0.483103239	0.046514435	0.983646501	0.547448624	0.868220298	0.000452408
TRUE	mDC	CD279	0.077195331	0.000966248	0.2400739	0.1933617	0.120357036	0.012194243
TRUE	mDC	CD57	0.045528806	0.027240821	0.63545146	0.747450162	0.531618957	0.01513697
TRUE	mDC	CD69	0.086696924	0.001085161	0.2499014	0.126220131	0.28398618	0.001380676
TRUE	mDC	IFNb	0.301446075	0.091217369	0.723952324	0.451097737	0.428968578	0.121266087
TRUE	mDC	IFNg	0.021443615	3.04E-05	0.333406656	0.090170514	0.429797346	4.62E-06
TRUE	mDC	IL6	0.050530665	0.000193537	0.421772691	0.171177155	0.270963733	0.000352534
TRUE	mDC	MIP1b	0.030816468	0.000101079	0.227087246	0.124968313	0.250949265	1.41E-05
TRUE	mDC	Perforin	0.042570458	0.002050098	0.726346106	0.670670322	0.188751183	0.00019715
TRUE	mDC	TNFa	0.114413041	0.008186996	0.579119276	0.665393522	0.751833055	0.009622858
FALSE	BN	CD152	0.265225708	0.914675249	0.298722585	0.392929403	0.347006275	0.365623082
FALSE	BN	CD279	0.692920319	0.240448727	0.519882969	0.656622506	0.361189845	0.607757832
FALSE	BN	CD57	0.068706466	0.74425159	0.248194012	0.878016528	0.469300087	0.727845985
FALSE	BN	CD69	0.7859503	0.363917838	0.655807574	0.97729341	0.866932271	0.493647931
FALSE	BN	IFNb	0.624755605	0.666790315	0.909480309	0.753860009	0.584461065	0.07339102
FALSE	BN	IFNg	0.613173195	0.450294422	0.829761875	0.967282509	0.453037587	0.028837029
FALSE	BN	IL6	0.22371425	0.268395223	0.563628727	0.9985738	0.874515155	0.044362786
FALSE	BN	MIP1b	0.978443995	0.127645001	0.976121406	0.836571227	0.89616758	0.040316165
FALSE	BN	Perforin	0.872764452	0.830129188	0.906872434	0.752824239	0.327900112	0.03280456
FALSE	BN	TNFa	0.206706626	0.790811976	0.945938421	0.615567386	0.515226674	0.921040533
FALSE	CD4+TN	CD152	0.961354686	0.754236899	0.786486071	0.137140067	0.180373567	0.039034712

FALSE	CD4+TN	CD279	0.901164781	0.95606772	0.351611064	0.970072141	0.261680451	0.733934533
FALSE	CD4+TN	CD57	0.451340991	0.531517415	0.652515016	0.218888889	0.895547405	0.414464739
FALSE	CD4+TN	CD69	0.696457867	0.917566648	0.591453792	0.389439964	0.274589769	0.188229226
FALSE	CD4+TN	IFNb	0.631744642	0.341211585	0.803101594	0.171605192	0.447750876	0.039439947
FALSE	CD4+TN	IFNg	0.507780497	0.82502017	0.785090875	0.189289353	0.018182994	0.003131619
FALSE	CD4+TN	IL6	0.928800337	0.995759459	0.819758995	0.214654741	0.169309353	0.038307382
FALSE	CD4+TN	MIP1b	0.896375429	0.810193971	0.908418191	0.236251279	0.071024242	0.008455557
FALSE	CD4+TN	Perforin	0.702776346	0.803709346	0.990676232	0.226868484	0.208095935	0.036712297
FALSE	CD4+TN	TNFa	0.619743312	0.817226224	0.77251367	0.172002044	0.45420226	0.0913081
FALSE	CD8+TN	CD152	0.214657682	0.937681143	0.93697884	0.701825849	0.044318372	0.350847568
FALSE	CD8+TN	CD279	0.187509271	0.908781615	0.863926264	0.658608925	0.093284191	0.801769558
FALSE	CD8+TN	CD57	0.622722097	0.517127552	0.370862785	0.812120814	0.540927255	0.139131942
FALSE	CD8+TN	CD69	0.041602006	0.635102253	0.492068294	0.924310599	0.044182657	0.417767524
FALSE	CD8+TN	IFNb	0.105014784	0.903123387	0.720433214	0.794652731	0.079129736	0.691877812
FALSE	CD8+TN	IFNg	0.211859143	0.573931715	0.854222743	0.9563499	0.07061414	0.468030164
FALSE	CD8+TN	IL6	0.136701766	0.645729674	0.932904418	0.912702097	0.069577841	0.347252355
FALSE	CD8+TN	MIP1b	0.123319632	0.984834139	0.820168499	0.911579166	0.040768923	0.348984648
FALSE	CD8+TN	Perforin	0.441237169	0.945403609	0.679351007	0.997952524	0.113084839	0.735531415
FALSE	CD8+TN	TNFa	0.062373814	0.484899999	0.580261731	0.928220881	0.032436035	0.258136969
FALSE	TregN	CD152	0.507604801	0.550869251	0.593902319	0.982202996	0.094882815	0.346995304
FALSE	TregN	CD279	0.87405637	0.548381639	0.544955728	0.524674229	0.102284268	0.628583609
FALSE	TregN	CD57	0.102814569	0.446375034	0.220282499	0.575569045	0.001055139	0.740259828
FALSE	TregN	CD69	0.267501245	0.975581452	0.482794909	0.588006023	0.053881808	0.681533287
FALSE	TregN	IFNb	0.023120944	0.127017623	0.144009011	0.759510259	0.013286876	0.217024694
FALSE	TregN	IFNg	0.152186054	0.134929498	0.174346512	0.708361712	0.007461201	0.733453598
FALSE	TregN	IL6	0.354600516	0.424759214	0.633350276	0.678186082	0.022356173	0.558970477
FALSE	TregN	MIP1b	0.110796381	0.273578314	0.211127185	0.585073749	0.001630092	0.797101419
FALSE	TregN	Perforin	0.057316162	0.824607	0.174512252	0.964404588	0.001213559	0.843500191
FALSE	TregN	TNFa	0.056104693	0.99905146	0.122731921	0.812479712	0.002270937	0.960121177
TRUE	CD16+Mono	CD152	0.633782866	0.210068842	0.599677157	0.837124581	0.114771895	0.241652347
TRUE	CD16+Mono	CD279	0.430853319	0.16629931	0.944616118	0.355210689	0.400505009	0.31298898
TRUE	CD16+Mono	CD57	0.538060026	0.865018035	0.848159492	0.582999558	0.078053448	0.594932177
TRUE	CD16+Mono	CD69	0.985324674	0.116051756	0.763618466	0.482736711	0.156918562	0.313364991
TRUE	CD16+Mono	IFNb	0.082932794	0.107660335	0.514371523	0.64536062	0.232678899	0.033647601
TRUE	CD16+Mono	IFNg	0.410079463	0.165541582	0.816144542	0.4945713	0.043194935	0.538113112
TRUE	CD16+Mono	IL6	0.169006584	0.165362044	0.644721875	0.711431315	0.037203834	0.436144847
TRUE	CD16+Mono	MIP1b	0.631713741	0.042985909	0.906700142	0.469421627	0.076254323	0.337893361
TRUE	CD16+Mono	Perforin	0.206577533	0.886798274	0.270209326	0.741498463	0.053217335	0.146504612
TRUE	CD16+Mono	TNFa	0.167303899	0.272537725	0.40521079	0.346010593	0.27696762	0.204900425
TRUE	NK	CD152	0.413058038	0.3413852	0.59873062	0.953017628	0.702641212	0.109101424
TRUE	NK	CD279	0.910482263	0.209008924	0.917166076	0.703559105	0.568345188	0.115688382
TRUE	NK	CD57	0.64839082	0.695608245	0.98557684	0.483818354	0.27271054	0.142987912
TRUE	NK	CD69	0.956094035	0.133481265	0.818264375	0.570100793	0.768831863	0.04960981
TRUE	NK	IFNb	0.209456057	0.134393913	0.908875073	0.616417823	0.83141131	0.065080612
TRUE	NK	IFNg	0.690949778	0.009172046	0.829061999	0.418896787	0.592578491	0.006952956
TRUE	NK	IL6	0.706021285	0.045256611	0.866153492	0.538950896	0.817622309	0.044205572
TRUE	NK	MIP1b	0.839216498	0.014625991	0.934848527	0.382674855	0.514488332	0.019767211
TRUE	NK	Perforin	0.222126385	0.765916808	0.726799753	0.865551417	0.726512088	0.74387479
TRUE	NK	TNFa	0.46550044	0.178622387	0.994260172	0.788789886	0.960595938	0.09982871
FALSE	BPb	CD152	0.434992774	0.988755944	0.231428392	0.42456161	0.894830243	0.055928557
FALSE	BPb	CD279	0.27785228	0.658073638	0.927365057	0.768863583	0.545562831	0.951460332
FALSE	BPb	CD57	0.623521141	0.050129635	0.642665598	0.444579398	0.991995755	0.974043555
FALSE	BPb	CD69	0.842615924	0.776663616	0.730515137	0.953206816	0.503625262	0.334163757
FALSE	BPb	IFNb	0.907472894	0.69372411	0.997656682	0.899076734	0.805632145	0.099886323
FALSE	BPb	IFNg	0.754528401	0.623535168	0.385091273	0.758423449	0.349869147	0.001306343
FALSE	BPb	IL6	0.849621851	0.380033246	0.214211051	0.923810155	0.322083304	0.003842866
FALSE	BPb	MIP1b	0.417007413	0.305836588	0.506378968	0.582076053	0.257783386	0.002998894
FALSE	BPb	Perforin	0.779927934	0.513263087	0.464817123	0.703095848	0.666197501	0.302746244
FALSE	BPb	TNFa	0.160540775	0.794441447	0.899355832	0.763399129	0.983450724	0.711670557
TRUE	pDC	CD152	0.650080416	0.175152911	0.642279296	0.504477069	0.35220269	0.017482493
TRUE	pDC	CD279	0.482497538	0.177490658	0.294786747	0.353681557	0.258335757	0.161748588

Zhao et al Table S3

TRUE	pDC	CD57	0.203750591	0.005773443	0.704243889	0.393367926	0.245093421	0.023969989
TRUE	pDC	CD69	0.043666222	0.018312978	0.242676771	0.244540327	0.529614396	0.323091979
TRUE	pDC	IFNb	0.082500055	0.140070782	0.667354047	0.645362473	0.047277417	0.053662936
TRUE	pDC	IFNg	0.310959331	0.309147004	0.923326255	0.734176239	0.033895511	0.030388019
TRUE	pDC	IL6	0.920673869	0.209852824	0.919437881	0.718236571	0.161511431	0.129381237
TRUE	pDC	MIP1b	0.253713207	0.052704597	0.539904499	0.553514006	0.568363425	0.050966836
TRUE	pDC	Perforin	0.076820187	0.129050581	0.521026883	0.823644893	0.478003251	0.422427451
TRUE	pDC	TNFa	0.329504207	0.25973343	0.70197429	0.714027556	0.021056733	0.037657489
FALSE	Th1	CD152	0.559491765	0.728712552	0.548197197	0.513211035	0.474347354	0.093506306
FALSE	Th1	CD279	0.308978403	0.997597014	0.85627754	0.958927489	0.019518208	0.094632684
FALSE	Th1	CD57	0.273994698	0.235481163	0.632091718	0.909274547	0.107274903	0.160768587
FALSE	Th1	CD69	0.342980189	0.2661846	0.53670121	0.709860537	0.127422612	0.037532398
FALSE	Th1	IFNb	0.902118699	0.225634591	0.758864204	0.917636148	0.310131137	0.012002543
FALSE	Th1	IFNg	0.84569907	0.360981669	0.767861804	0.920900247	0.773818682	0.005462874
FALSE	Th1	IL6	0.954116268	0.254834654	0.753097851	0.928359379	0.930240688	0.017804181
FALSE	Th1	MIP1b	0.731369158	0.166914319	0.880146611	0.818406965	0.955673184	0.021429521
FALSE	Th1	Perforin	0.831288986	0.150334947	0.857366944	0.879470301	0.205698703	0.026456697
FALSE	Th1	TNFa	0.616366732	0.021539333	0.792453412	0.753853224	0.067753986	0.007523591
FALSE	Th17	CD152	0.636604229	0.316641325	0.226810546	0.55592509	0.476494033	0.013918908
FALSE	Th17	CD279	0.739201332	0.91815609	0.678942662	0.773163124	0.08850193	0.108416773
FALSE	Th17	CD57	0.06265259	0.129773059	0.062456335	0.962364358	0.032926671	0.062092355
FALSE	Th17	CD69	0.289376384	0.118438259	0.583750114	0.820813481	0.118270601	0.060391112
FALSE	Th17	IFNb	0.151663952	0.2508571	0.457172054	0.767748388	0.087748971	0.072091595
FALSE	Th17	IFNg	0.872852772	0.206877637	0.280067361	0.824258908	0.296756898	0.058925657
FALSE	Th17	IL6	0.823890576	0.409119416	0.20079866	0.663543428	0.339322466	0.063246856
FALSE	Th17	MIP1b	0.654599404	0.118982107	0.525829016	0.901617751	0.30488877	0.029287802
FALSE	Th17	Perforin	0.372581783	0.185731532	0.609258645	0.836498265	0.114060968	0.074136276
FALSE	Th17	TNFa	0.272780377	0.215806857	0.788300623	0.777363709	0.192958238	0.123173487
FALSE	Non-Th1/17	CD152	0.127334373	0.790408942	0.211198647	0.494589443	0.735483725	0.011987154
FALSE	Non-Th1/18	CD279	0.062763555	0.744087046	0.617361302	0.973368751	0.571838712	0.164983776
FALSE	Non-Th1/19	CD57	0.359364	0.015740128	0.811280417	0.801265919	0.072558403	0.200211063
FALSE	Non-Th1/20	CD69	0.347807787	0.206775776	0.825775447	0.78330589	0.354795674	0.057313207
FALSE	Non-Th1/21	IFNb	0.42639094	0.491329546	0.8002049	0.965041877	0.374800182	0.081083597
FALSE	Non-Th1/22	IFNg	0.075054868	0.222565887	0.274919925	0.968721155	0.610783829	0.004043619
FALSE	Non-Th1/23	IL6	0.0415082	0.261783623	0.224188288	0.916843143	0.628437903	0.026042946
FALSE	Non-Th1/24	MIP1b	0.074815277	0.1256472	0.368835952	0.930603685	0.654578098	0.00865698
FALSE	Non-Th1/25	Perforin	0.154411716	0.29901792	0.412257829	0.89859308	0.995941263	0.009520531
FALSE	Non-Th1/26	TNFa	0.090858016	0.055849813	0.206621658	0.868907108	0.851632906	0.048549342
FALSE	BTrans	CD152	0.539630837	0.308423561	0.732990687	0.524407932	0.115839945	0.023488065
FALSE	BTrans	CD279	0.500713563	0.828035379	0.988808227	0.918239488	0.477446049	0.451189479
FALSE	BTrans	CD57	0.334194606	0.553023657	0.609365301	0.883087519	0.429357419	0.800306085
FALSE	BTrans	CD69	0.968960276	0.351416416	0.890090319	0.779795578	0.005968382	0.031227221
FALSE	BTrans	IFNb	0.370481396	0.021797556	0.983411179	0.765413669	0.179048934	0.007484332
FALSE	BTrans	IFNg	0.746959309	0.276844171	0.973403976	0.992068542	0.013259032	0.007168834
FALSE	BTrans	IL6	0.445000909	0.324895844	0.807767837	0.997978096	0.041274321	0.026411556
FALSE	BTrans	MIP1b	0.641646806	0.200095768	0.888597094	0.971066215	0.052711085	0.00913537
FALSE	BTrans	Perforin	0.479944917	0.60079183	0.562181018	0.893261425	0.212445583	0.30342192
FALSE	BTrans	TNFa	0.674910182	0.874517363	0.854770146	0.620848989	0.204662619	0.191819491
FALSE	Treg	CD152	0.062257368	0.540545124	0.30374425	0.837209277	0.052285239	0.617280027
FALSE	Treg	CD279	0.368629815	0.351737989	0.149088509	0.779684775	0.243415892	0.353655744
FALSE	Treg	CD57	0.011839619	0.510082626	0.046159844	0.94745597	0.048552398	0.876715893
FALSE	Treg	CD69	0.140860213	0.831804985	0.21136788	0.692600406	0.125342769	0.718251102
FALSE	Treg	IFNb	0.006420635	0.082096988	0.032973525	0.460085363	0.021000458	0.31182157
FALSE	Treg	IFNg	0.022890666	0.994600346	0.071464947	0.864470639	0.032182078	0.998747558
FALSE	Treg	IL6	0.057510744	0.528674661	0.106678809	0.98064255	0.076358819	0.76939419
FALSE	Treg	MIP1b	0.020481796	0.769460559	0.095231214	0.826588457	0.01384374	0.719151858
FALSE	Treg	Perforin	0.00281805	0.196810368	0.044522881	0.943556378	0.019934146	0.489714301
FALSE	Treg	TNFa	0.004530869	0.236972449	0.027695873	0.822226459	0.015899633	0.644298534
